# European Health Data & Evidence Network—learnings from building out a standardized international health data network

**DOI:** 10.1093/jamia/ocad214

**Published:** 2023-11-10

**Authors:** Erica A Voss, Clair Blacketer, Sebastiaan van Sandijk, Maxim Moinat, Michael Kallfelz, Michel van Speybroeck, Daniel Prieto-Alhambra, Martijn J Schuemie, Peter R Rijnbeek

**Affiliations:** OHDSI Collaborators, Observational Health Data Sciences and Informatics (OHDSI), New York, NY, United States; Department of Medical Informatics, Erasmus University Medical Center, Rotterdam, the Netherlands; Janssen Pharmaceutical Research and Development LLC, Raritan, NJ 08869, United States; OHDSI Collaborators, Observational Health Data Sciences and Informatics (OHDSI), New York, NY, United States; Department of Medical Informatics, Erasmus University Medical Center, Rotterdam, the Netherlands; Janssen Pharmaceutical Research and Development LLC, Raritan, NJ 08869, United States; OHDSI Collaborators, Observational Health Data Sciences and Informatics (OHDSI), New York, NY, United States; Odysseus Data Services, Prague, Czech Republic; OHDSI Collaborators, Observational Health Data Sciences and Informatics (OHDSI), New York, NY, United States; Department of Medical Informatics, Erasmus University Medical Center, Rotterdam, the Netherlands; OHDSI Collaborators, Observational Health Data Sciences and Informatics (OHDSI), New York, NY, United States; Odysseus Data Services, Prague, Czech Republic; Janssen Pharmaceutical Research and Development LLC, Raritan, NJ 08869, United States; OHDSI Collaborators, Observational Health Data Sciences and Informatics (OHDSI), New York, NY, United States; Department of Medical Informatics, Erasmus University Medical Center, Rotterdam, the Netherlands; Centre for Statistics in Medicine, NDORMS, University of Oxford, Oxford, United Kingdom; OHDSI Collaborators, Observational Health Data Sciences and Informatics (OHDSI), New York, NY, United States; Janssen Pharmaceutical Research and Development LLC, Raritan, NJ 08869, United States; Department of Biostatistics, University of California, Los Angeles, CA 90095, United States; OHDSI Collaborators, Observational Health Data Sciences and Informatics (OHDSI), New York, NY, United States; Department of Medical Informatics, Erasmus University Medical Center, Rotterdam, the Netherlands

**Keywords:** OMOP common data model, observational data, data standardization

## Abstract

**Objective:**

Health data standardized to a common data model (CDM) simplifies and facilitates research. This study examines the factors that make standardizing observational health data to the Observational Medical Outcomes Partnership (OMOP) CDM successful.

**Materials and methods:**

Twenty-five data partners (DPs) from 11 countries received funding from the European Health Data Evidence Network (EHDEN) to standardize their data. Three surveys, DataQualityDashboard results, and statistics from the conversion process were analyzed qualitatively and quantitatively. Our measures of success were the total number of days to transform source data into the OMOP CDM and participation in network research.

**Results:**

The health data converted to CDM represented more than 133 million patients. 100%, 88%, and 84% of DPs took Surveys 1, 2, and 3. The median duration of the 6 key extract, transform, and load (ETL) processes ranged from 4 to 115 days. Of the 25 DPs, 21 DPs were considered applicable for analysis of which 52% standardized their data on time, and 48% participated in an international collaborative study.

**Discussion:**

This study shows that the consistent workflow used by EHDEN proves appropriate to support the successful standardization of observational data across Europe. Over the 25 successful transformations, we confirmed that getting the right people for the ETL is critical and vocabulary mapping requires specific expertise and support of tools. Additionally, we learned that teams that proactively prepared for data governance issues were able to avoid considerable delays improving their ability to finish on time.

**Conclusion:**

This study provides guidance for future DPs to standardize to the OMOP CDM and participate in distributed networks. We demonstrate that the Observational Health Data Sciences and Informatics community must continue to evaluate and provide guidance and support for what ultimately develops the backbone of how community members generate evidence.

## Introduction

Health data comes in many forms: electronic health records (EHRs) (eg, general practitioner [GP]), clinical registries, longitudinal survey data, insurance claims data, and much more. Health data is often collected and stored in different ways which makes standardized research across data from multiple institutions difficult. Research can be challenging even within a single institution where multiple data sources are used. Conversion of health data to a common data model (CDM) facilitates research by transforming the data into a common format with a standardized vocabulary. This standardization allows for systematic analysis across disparate health data sources.^1^ The Observational Health Data Sciences and Informatics (OHDSI)^2^ community, a multi-stakeholder, interdisciplinary collaborative that strives to bring value out of health data through large-scale analytics, relies on its CDM model called Observational Medical Outcomes Partnership (OMOP) CDM.^3^ This person-centric model is the backbone of how OHDSI improves observational research to produce a comprehensive understanding of health and disease.^4^

The process of standardizing health data to the OMOP CDM is referred to as the extract, transform, and load (ETL) process. The Book of OHDSI^4^ suggests 4 main steps for this process: data and CDM experts design the ETL, individuals with medical knowledge map source vocabulary codes to standardized codes, a technical person implements the ETL, and a quality control process is implemented. The OHDSI community created open-source tools for these steps.^5–9^ Building and performing an ETL to the OMOP CDM is an investment, but the return on that investment is ready access to sophisticated analytics and ability to participate in network studies.

Even with process recommendations and supporting tools, building an ETL for any CDM can be challenging for some teams. Ong et al.^2^ identified 24 technical hurdles that often arise throughout an ETL process; including challenges working with source data, technical difficulties, issues with knowledge management, code management and versioning issues, data quality concerns, and ETL operation challenges. Improving the ETL process starts with understanding these problems. While the OHDSI community has recommendations for developing an ETL, the challenges of developing one have never been formally evaluated.

During the coronavirus disease pandemic (COVID-19) crisis, the European Health Data & Evidence Network (EHDEN),^10^ a public-private partnership with a goal to build a large-scale, federated network of health data standardized to the OMOP CDM, held a call for data. The COVID-19 Rapid Collaboration Call, or EHDEN data call, invited institutions to apply for financial and technical support to standardize their data that included COVID-19 patients.^11^ The goal was to produce high-quality standardized data to support important characterizations of patients with COVID-19, learn how to best manage their care, and ensure their treatments are safe and effective.^11^ This work is of particular importance in Europe, as converting to the OMOP CDM helps address the most important challenge in international projects, interoperability of different data sources and the difference in terminology, by creating a research environment leveraging federated data sources. Twenty-five data partners (DPs) were awarded the grant. This EHDEN data call provided a unique opportunity to understand key success factors for the development of an ETL, where success can be defined as both a timely development of an ETL and network research involvement.

This work aims to evaluate the critical factors that contribute to the success of standardizing health data to the OMOP CDM. Success was evaluated based on the timeliness of developing the ETL and the ability to participate in network research. To capture these measurements, from each DP, we collected data about their journey to the OMOP CDM as well as surveyed them about any ETL challenges they faced. By understanding what factors lead to a successful transformation of a data source to the OMOP CDM, we will be able to provide further recommendations for a preferred ETL process.

## Methods

### COVID-19 Rapid Collaboration Call

Twenty-five DPs representing 11 different countries were awarded financial support to standardize their data to the OMOP CDM under the EHDEN data call.^11^ The 11 countries included Belgium, Denmark, Estonia, France, Italy, Portugal, Serbia, Spain, the Netherlands, Turkey, and the United Kingdom. The size of the databases ranged from 400 up to 39 million persons, representing a range of different data types including hospital (inpatient only), EHRs (mix of inpatient and outpatient data), claims, and registry data. Details about each database can be found in Appendix S1 and was sourced from the publicly available EHDEN Catalog (https://portal.ehden.eu/).

**Figure 1.**
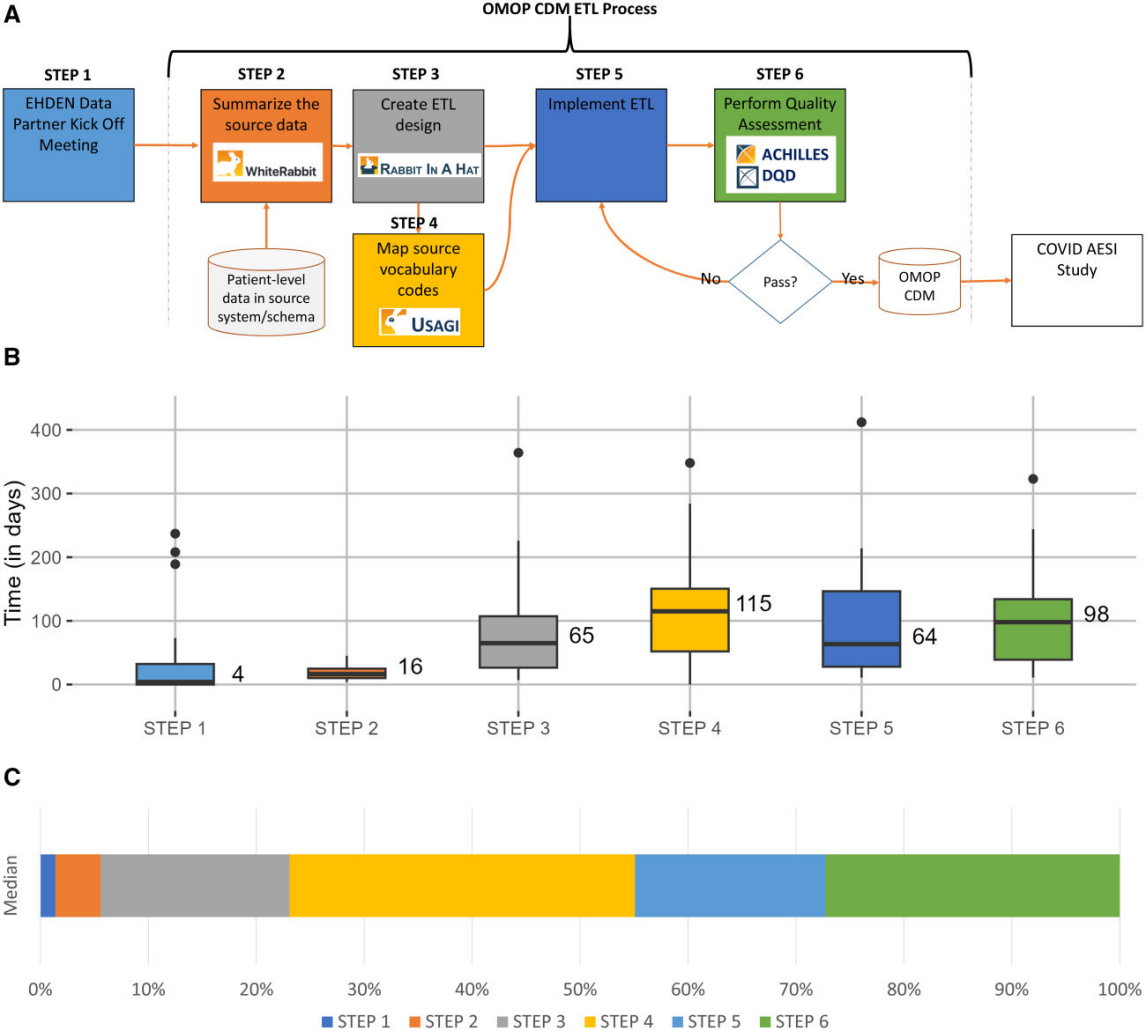
OMOP CDM ETL development process: (A) represents the ETL process map, (B) is a box plot of median length in days for each step across all data partners, and (C) is a stacked bar chart showing the percentage of median time each step took. CDM, common data model; COVID AESI Study, “Adverse Events of Special Interest within COVID-19 Subjects” study; DQD, DataQualityDashboard; EHDEN, European Health Data & Evidence Network; ETL, extract, transform, and load; OMOP, outcomes partnership common data model.

Each DP was expected to follow the current OMOP CDM ETL development process suggested by the OHDSI community, as seen in Figure 1A. They started by summarizing their source data using a tool called White Rabbit.^5^^,^^12^ The output of this step allowed the teams to learn about their data as well as use a tool called Rabbit-in-a-Hat, a graphical user interface facilitating the collaborative design of the ETL. In some cases, the Usagi tool was used to facilitate the mapping of source vocabularies to standard terminologies.^6^^,^^13^ Once this work was done, the DP was ready to implement their ETL. In this step, DPs chose the tools and methods best suited to their institution to execute the ETL. Once the data was transformed, the resulting database was evaluated using the ACHILLES and DataQualityDashboard (DQD) tools.^7^^,^^8^ Any issues discovered using these tools were addressed and incorporated back either into the ETL or the vocabulary mapping steps. The ETL process was re-executed and re-evaluated for quality. The goal of this iterative process was to produce a research-ready CDM database. These tools and processes are described in more detail in the Book of OHDSI.^4^

The EHDEN data call diverged from the regular OMOP ETL process in 3 ways. First, each DP, except one, was paired with the EHDEN Taskforce to facilitate the conversion; the EHDEN Taskforce were ETL specialists. The excluded DP knew the OMOP ETL procedure and did not need support. Second, once DPs had completed their ETLs they were offered the opportunity to participate in research initiatives. Specifically, some of the DPs participated in the study “Adverse Events of Special Interest (AESI) within COVID-19 Subjects,” or the *COVID-19 AESI study* for short, designed to estimate background rates of adverse events after COVID-19 disease.^14–16^ This allowed DPs to exercise their data in their new OMOP CDM and obtain network-based research skills. The third and final significant change was that the DPs were requested to complete surveys at various points in the ETL process, allowing us to collect data that might assist in better understanding the critical success elements.

### Survey development

Three surveys, found in Appendix S2, were created with a mix of open-ended and multiple-choice questions. The survey questions were developed with the Framework for ETL Challenge Classification outlined by Ong et al.^2^ The themes of the framework were divided across the surveys: Survey 1 covered source data; Survey 2 covered technical difficulties and knowledge management; and Survey 3 covered code management and versioning, data quality, and ETL operations. The survey questions were refined based on feedback from 4 individuals who had experience both with the OMOP CDM and EHDEN. Google Forms (https://docs.google.com/forms), a free survey tool, was used to collect responses. Every member associated with the DP team was able to complete the survey, meaning it was possible for multiple replies per survey for each DP.

### Data collection

Four types of data were collected. First, the 3 surveys were given as DPs progressed through their ETL. Survey 1 was given after the project kickoff to each DP, Survey 2 was given after the ETL design was completed, and Survey 3 was given at the completion of the OMOP CDM. Survey 1 was taken for the first time on May 13, 2020, and Survey 3 was taken for the last time on May 17, 2022. Since DPs could start and stop their projects at their own rate, progress was fluid during those 2 years.

Data associated with the ETL progress were also collected during regular meetings with DPs. For example, the start and stop of each step were tracked, as illustrated in Figure 1A, notes from meetings, as well as a list of vocabularies found within each data source.

The third type of data collected was the results from running DQD. This was already part of the process each DP needed to go through to complete their work in the EHDEN data call. The number of checks runs in DQD and the outstanding issues were reviewed by the EHDEN Taskforce.

Finally, it was recorded whether the DP participated in the *COVID-19 AESI study*.^16^

### Analysis

Our measures of success were the total number of days to transform source data into the OMOP CDM and if a DP participated successfully in network research. DPs were successful if they took less than 365 days to complete their transformation and were part of the *COVID-19 AESI study*.^16^ The duration of 365 days was selected because the EHDEN contract stipulated that work should finish within that time. Additionally, in order to understand the association between our key measures of success and the survey responses, we reported the results as bar charts dichotomized by the outcome measures. Depending on the survey question, answers were either summarized as max per DP (max was chosen as a convenient and consistent way to summarize) or summarized across all answers provided by members of the DP. The summary tactics used are discussed in the “Results” section.

## Results

In total, 25 DPs participated in this EHDEN data call, representing 11 different countries, collectively covering more than 67 million patient records from claims, EHR, and registries from GP, secondary care, and hospital systems. This represents the largest cross-sectional view of European data to date and is only 13% of the DPs participating in the EHDEN consortium (as of April 2023). Information about the 25 DP’s databases can be found in Table 1 with details in Appendix S1.

**Table 1.** COVID-19 rapid collaboration call data partners information with participation in EHDEN taskforce, the 3 surveys, and network research study as of August 2022.

Short name	Full name	Patient count	Country	Type	Task-force	Survey			Completed call for data	Participated in study
						1	2	3		
APHM	Health Data Warehouse of Assistance Publique—Hopitaux de Marseille	2.47 M	France	Hospital data	Yes	Yes	Yes	Yes	Yes	Yes
APUM^a^	Azienda Policlinico Universitaria di Modena	400	Italy	EHR	Yes	Yes	Yes	*No*	*No*	*No*
AUMC	Pacmed Data Warehouse at Amsterdam University Medical Center	1.83 k	The Netherlands	Hospital data	Yes	Yes	Yes	Yes	Yes	*No*
BIOCRUCES	Biocruces Bizkaia Health Research Institute	45 k	Spain	EHR	Yes	Yes	Yes	Yes	Yes	*No*
CC_NIS	University Clinical Center Nis	223 k	Serbia	EHR	Yes	Yes	*No*	Yes	Yes	*No*
CC_SERBIA	University Clinical Center of Serbia	823 k	Serbia	EHR	Yes	Yes	Yes	Yes	Yes	Yes
CPRD_AURUM	Clinical Practice Research Datalink—AURUM+Hospital Episode Statistics Admitted Patient Care (HES APC) data	39 M	United Kingdom	GP	Yes	Yes	Yes	Yes	Yes	Yes
CSS	Center for Surgical Science	76.8 k	Denmark	Registry	Yes	Yes	Yes	Yes	Yes	*No*
DATALOCH^a^	DataLoch	414 k	United Kingdom	EHR	Yes	Yes	Yes	Yes	Yes	*No*
FIIBAP^a^	Fundación para la Investigación e Innovación Biosanitaria en Atención Primaria	292 k	Spain	EHR	Yes	Yes	Yes	Yes	Yes	Yes
FINCB^a^	Fondazione IRCCS Istituto Neurologico Carlo Besta	766	Italy	Hospital data	Yes	Yes	Yes	Yes	Yes	*No*
FPIO^a^	Fondazione Poliambulanza Istituto Ospedaliero	23.1 k	Italy	EHR	Yes	Yes	Yes	Yes	Yes	*No*
HDH^a^	Health Data Hub	91.9 k	France	EHR	Yes	Yes	Yes	*No*	*No*	*No*
HIC	Health Informatics Centre	1.3 M	United Kingdom	EHR	Yes	Yes	Yes	Yes	Yes	Yes
IDIVAL	Servicio Cántabro de Salud and IDIVAL	580 k	Spain	EHR	Yes	Yes	Yes	Yes	Yes	*No*
IMASIS	Parc de Salut Mar Barcelona Information System (IMASIS)	976 k	Spain	EHR	Yes	Yes	Yes	Yes	Yes	Yes
IRCCSE^a^	Azienda Unità Sanitaria Locale—IRCCS in Reggio Emilia	1.8 k	Italy	EHR	Yes	Yes	Yes	Yes	Yes	*No*
IU	Istanbul Faculty of Medicine, Istanbul University	899 k	Turkey	EHR	Yes	Yes	*No*	Yes	Yes	Yes
LYNXCARE	LynxCare Clinical Informatics	4 dbs (range 2.5 k-20 k)	Belgium	EHR	Yes	Yes	Yes	Yes	Yes	*No*
MEDAMAN	Medaman Hospital Data	117 k	Belgium	EHR	Yes	Yes	Yes	Yes	Yes	Yes
RCGP	Royal College of General Practitioners Research and Surveillance Centre	11 M	United Kingdom	EHR	Yes	Yes	Yes	*No*	*No*	*No*
SIDIAP	The Information System for Research in Primary Care	8 M	Spain	GP	Yes	Yes	Yes	Yes	Yes	Yes
U_OF_TARTU	University of Tartu	386 k	Estonia	Claims	*No*	Yes	*No*	*No*	Yes	Yes
UK_BIOBANK	UK Biobank	502 k	United Kingdom	Registry+EHR	Yes	Yes	Yes	Yes	Yes	Yes
ULSM^a^	Unidade Local de Saúde de Matosinhos	9.75 k	Portugal	EHR	Yes	Yes	Yes	Yes	Yes	*No*

a COVID-19 only datasets.

dbs, databases; EHR, electronic health record; GP, general practitioner.

### Survey results

As of August 2022, all 25 DPs completed Survey 1, while 88% completed Survey 2, and 84% Survey 3. Survey 1 had an average of 4.6 individuals from each DP respond (1 minimum—11 maximum). Both Survey 2 and Survey 3 had an average of 1.7 individuals from each DP respond (1 minimum—4 maximum). Twenty-two DPs completed all requirements of the call. Twenty-one DPs received support from the EHDEN Taskforce. Ultimately 21 DPs both completed the EHDEN data call and worked with an EHDEN Taskforce. Most of the results are reported for the 21 DPs and all results are specified if they are for the 21 DPs or for all 25 DPs. Ten (48%) of the 21 DPs participated in the *COVID-19 AESI Study*.

Table 2 summarizes key questions from Survey 1 for 21 DPs. The first question summarized was “What will be your primary role in this project?” For example, 20 out of 21 DPs had at least one person whose primary role in the project was “informatician.” Another question was “How would you classify your expertise with the data source?” Eleven DPs had at least one person who considered themselves an expert. Finally, for the question “Realistically, how many hours a week can you dedicate to this project?,” 6 DPs had at least one person planning on spending 9-16 h/week on the EHDEN data call.

**Table 2.** Summary of key questions in survey 1 for 21 data partners (that were both completed with the COVID-19 rapid collaboration call and worked with the EHDEN taskforce).

Survey question	Options	No. of data partners
What will be your primary role in this project? *(summarized as often as the role showed up, it is possible for multiple roles to be present on one team, however the role was only counted once per team)*	Informatician	20
	Computer scientist	17
	Project manager	16
	Data manager	8
	Clinical scientist	7
	Person in medicine	4
	Health policy individual	3
	Epidemiologist	3
	Statistician	2
	Something other than the above	5
	Preferred not to say	0
How would you classify your expertise with the data source? *(summarized max per data partner, every data partner counted once)*	Novice (minimal knowledge of the data source)	0
	Beginner (working knowledge of the data source)	2
	Competent (good working knowledge of the data source)	5
	Proficient (in depth understanding of the data source)	3
	Expert (authoritative knowledge of data source)	11
Realistically, how many hours a week can you dedicate to this project? *(summarized max per data partner, every data partner counted once)*	0-4 h/week	2
	5-8 h/week	5
	9-16 h/week	6
	17-24 h/week	3
	25-32 h/week	2
	33-40 h/week	3

Table 3 summarizes key questions from Survey 2 for the 21 of which 90% responded. When the DPs were asked “How many tables from your source data are in your ETL?” (max number by DP), 29% reported having less than 10 tables, 33% reported having 10 or more tables, and 38% did not reply. When asked “Thinking of your source data’s tables being converted to the OMOP CDM, prior to starting this Rapid Collaboration Call how much experience did you or your team have with these tables needed in the ETL?” (max choice of most familiar selected by DP), 52% reported being familiar with most or all of the tables, 38% reported being familiar with none, few, or some of the tables, and 10% did not reply. When asked “To use your source data in this Rapid Collaboration Call was there any effort necessary to prepare the data?” (max choice of most effort selected by DP), all DPs that replied to the survey said there was some effort to prepare the data (38% said there was much effort and 52% said there was some effort needed). When asked “Prior to the Rapid Collaboration call were you familiar with the OMOP Common Data Model?” (max choice of most familiar selected by DP), 57% said they were familiar. Finally, when asked, “Have you learned anything about your source data by going through this process?” (max choice of someone learning was selected by DP), 62% responded “Yes,” 24% responded “No,” 5% responded “Based on my involvement I cannot comment.,” and 10% did not answer.

**Table 3.** Summary of key questions in survey 2 for 21 data partners (that were both completed with the COVID-19 rapid collaboration call and worked with the EHDEN taskforce).

Survey question	Options	No. of data partners
How many tables from your source data are in your ETL? *(selected the max number reported in survey, these numbers were then categorized into <10 tables and ≥10 tables)*	Less than 10 tables	6
	Greater than or equal to 10 tables	7
	No response reported	8
Thinking of your source data’s tables being converted to the OMOP CDM, prior to starting this Rapid Collaboration Call how much experience did you or your team have with these tables needed in the ETL? *(selected the max choice from data partner, the choices were dichotomized into two options)*	Familiar with none/few/some of the tables	8
	Familiar with most/all of the tables	11
	No response reported	2
To use your source data in this Rapid Collaboration Call was there any effort necessary to prepare the data? For example, did a data extract from source systems need to be put in place in order for data to be available for the ETL process? *(selected the max choice per data partner)*	Yes, much effort was needed to prepare the raw data for ETL.	8
	Yes, some effort was needed to prepare the raw data for ETL.	11
	No, the data was in a format prior to applying to the Rapid Collaboration Call that was suitable for ETL.	0
	No response reported	2
Prior to the Rapid Collaboration call were you familiar with the OMOP Common Data Model? *(selected the max choice per data partner)*	Yes	12
	No	7
	No response reported	2
Have you learned anything about your source data by going through this process?	Yes	13
	No	5
	Based on my involvement I cannot comment.	1
	No response reported	2

Table 4 summarizes key questions from Survey 3 for 21 DPs of which all had at least one response. When asked about the complexity of the ETL process (max level of difficulty reported by DP), 67% of DPs found the process easy or neutral in complexity. When asked about the effectiveness of the tools used to assess data quality (eg, ACHILLES, DQD) (selecting the least helpful choice by DP), 71% of DPs found the tools supportive but needed support from the EHDEN Taskforce to use them appropriately. Additionally, only 19% of DPs said that their organization had formal plans to improve their CDM and use it for research in the future, 57% said the organization still needs to better understand the value to continue moving forward, and 24% were not sure of the plans for the CDM moving forward (max reply of how confident the DP was that the CDM would be used was selected). Finally, the ETL step that was most challenging for DPs was mapping source vocabulary codes to standardized concepts (all unique answers were summarized).

**Table 4.** Summary of key questions in survey 3 for 21 data partners (that were both completed with the COVID-19 rapid collaboration call and worked with the EHDEN taskforce).

Survey question	Options	No. of data partners
Given now that you are at the end or near the end of this process, how complex did you find the ETL process was for your data source to be converted to the OMOP Common Data Model? *(selected the max choice from data partner, the choices were dichotomized into two options)*	01) Difficult	7
	02) Easy and neutral	14
There are several tools we used to assess data quality (ie, ACHILLES, Data Quality Dashboard, CDM Inspection Report, and Catalog Export). Please select the answer that best fits your experience: *(selected the max choice per data partner)*	Even with the tools, I did not feel like there were detailed specifications for what to assess. Even with the help from the EHDEN Task Force I did not find the tools helpful or informative.	0
	The tools were supportive, however without the EHDEN Task Force I am not sure if I would have been able to make sense of what to do with the information.	6
	The tools provided insight, and I would have been able to make improvements on my own (without the EHDEN Task Force) but it would have been difficult or slow.	9
	The tools were useful, with them alone I could have made most of the necessary improvements needed for my CDM.	3
	The tools provided an obvious way to assess data quality and I was clear how to interpret the results.	2
	Based on my involvement I cannot comment.	1
Please choose the statement that most accurately describes your organization’s view on maintaining the CDM moving forward: *(selected the max choice from data partner, the choices were simplified to 3 options)*	01) I am not aware of my organization’s thoughts on our CDM’s use moving forward.	5
	02) We have made our CDM, have some plans to update, and may still need to see the value.	12
	03) We have made our CDM and plan to improve upon it moving forward to facilitate our organization’s research on our medical data.	4
Please select which step in the ETL process you found the most challenging: *(all unique choices per data partner were selected, each response can be up to 21)*	Summarizing the source data with White Rabbit	2
	Create ETL design	2
	Mapping source vocabulary codes to standardized concepts	11
	Setting up an environment for the processing of the ETL	2
	Implement ETL	6
	Perform data quality assessment	5
	Other	3

### ETL process measures

Of the 21 DPs, the median time it took to complete the ETL process was 358 days, with the shortest time being 172 days and the longest being 622 days and an interquartile range of 276-481. Figure 1B illustrates the duration of each step in the ETL process. Figure 1C is a stacked bar chart illustrating which process steps consumed the most time (based on the median days). Step 4 (mapping vocabulary codes) and Step 6 (performing quality assessment) required the greatest time. To understand the reasons why individual steps took the time they did, the regular meeting notes were used to understand the details.

In Step 1, 1 DP required 237 days to begin working with their data, 3 DPs required more than 100 days, and 9 DPs experienced some delay. The cause for the delay was almost invariably a lack of appropriate staff or data access issues. Step 2 was the least complicated of the steps; it consisted of a WhiteRabbit database scan; however, 1 DP required 45 days to complete the assignment. This was because this DP’s data was dispersed over multiple systems, and it required some time to find the appropriate personnel to assist with the scan. Step 3, creating the ETL, took 1 DP 364 days to complete. The same DP also took the longest to complete Step 5, completing data quality evaluation, with 412 days. These processes took a long time since the project’s primary developer did not always have the required access or rights to complete the task, resulting in several technical obstacles over the duration of the project. In addition, the person most knowledgeable about the data was not always accessible to the lead developer; therefore, we frequently had to wait for the two to communicate in order to resolve issues. Step 4, vocabulary mapping, took 1 DP 348 days to complete due to the rearrangement of the team working on the process, which resulted in little to no work being performed for the majority of the time. Once the new team was assigned, work went rapidly. Many of the DPs that took the longest at each phase were affected by the availability of the appropriate personnel and access or readiness of data.

Of the 21 DPs, 20 had at least one source vocabulary that needed to be mapped to standardized terminologies using the OMOP Vocabulary or tools like Usagi. The remaining DP did not spend time mapping vocabularies as they were planning to adopt the same EHR platform as another DP and thus could leverage their mapping work. Among the 20 DPs, the median number of source vocabularies was 7, ranging from 1 to 28. The median number of these vocabularies that were not in the OMOP Vocabulary and required the DP to spend time linking source vocabularies to standard terminology was 3.5, ranging from 1 to 21. This means that DPs needed to map a median of 59% of their source vocabulary concepts, ranging from 13% to 100%.

### Results from DQD

DQD results were shared from 20 of the DPs. The failure to collect DQD files from a single DP was an oversight. The median number of times a DP ran the DQD was 3 with an interquartile range of 2-7. Step 6, which coupled DQD and ACHILLES review, took a median of 98 days that ranged between 11 and 323 days. The most common issues identified in the first run were related to how well the database conformed to the technical specifications of the OMOP CDM. As these were addressed, subsequent runs of the DQD revealed more complicated issues, typically related to the mapping of site-specific codes to the standardized vocabulary and vocabulary domain harmonization.^17^

### Success measures

**Figure 2.**
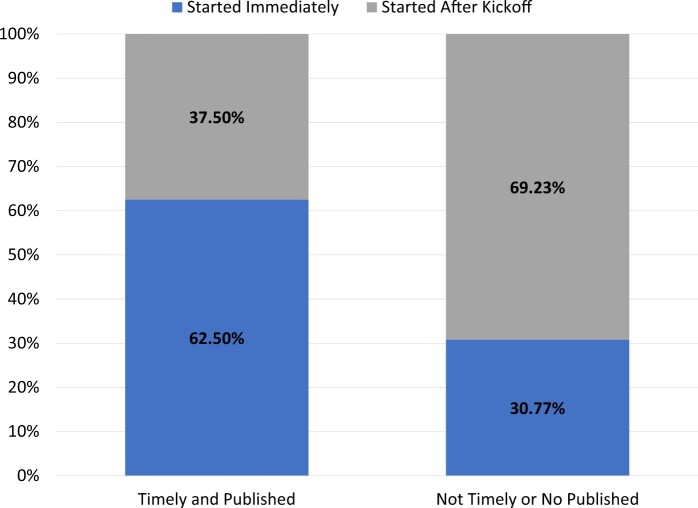
For data partners that met success criteria (timely and published, *n* = 8) versus those who did not meet the success criteria (not timely or not published, *n* = 13), how often did that data partner start immediately or after kick off.

**Figure 3.**
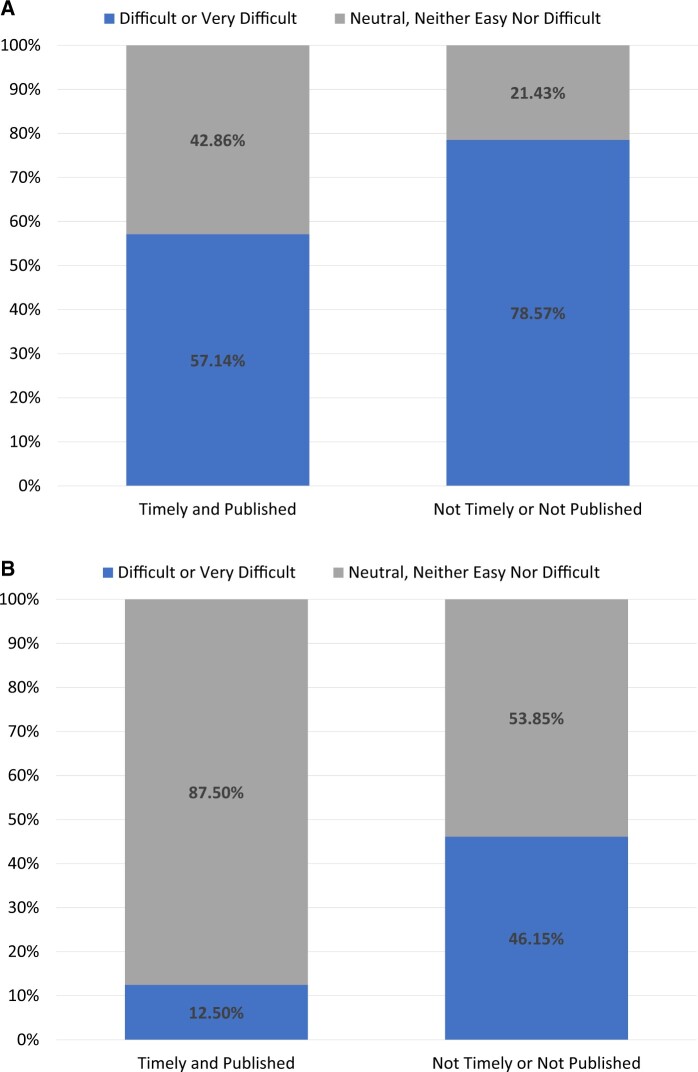
For data partners that met success criteria (timely and published, *n* = 8) versus those who did not meet the success criteria (not timely or not published, *n* = 13): (A) represents at the beginning of the process how difficult did they think the process would be and (B) represents at the end of the process how often did that data partner find the process difficult.

Of the 21 DPs, 52% had built their CDM in under 365 days, 48% participated in the *COVID-19 AESI study*,^16^ and 38% did both. A few correlations can be seen between the results and success markers. For instance, when evaluating how long it took a DP to start working on the project, Figure 2 demonstrates that 63% of those DPs who started right away (ie, the same day) met both success criteria as opposed to 31% of those who did not. Furthermore, DPs who thought the ETL process would be difficult before beginning the ETL were less likely to achieve the success indicators. DPs that did not achieve success 79% thought the process would be difficult versus 57% of DPs who did achieve success and thought the process would be difficult (Figure 3A). This trend persisted even after the process was completed (46% of the DPs who failed to meet the success indicators found the ETL process difficult, but only 13% of the DPs who met the success criteria did) (Figure 3B).

## Discussion

To the best of our knowledge, this is the most comprehensive assessment of the critical factors that contribute to the successful conversion of complex health-care data to the OMOP CDM. It was performed during the COVID-19 pandemic, a period when access to data was limited and team members were not continuously available. Nevertheless, most ETLs where realized within the agreed timelines. By doing this, all DPs were able to successfully improve the interoperability of their data which allowed them to participate in research studies using standardized analyses.

Our study identified multiple factors that had a major impact on timelines which were uncovered using the ETL measures captured. For example, studying the duration of tasks and reviewing themes among DPs that took a long time on certain steps allowed us to uncover the importance of establishing governance rules prior to the initiation of the work. Also, through both the surveys and ETL measures captured, we learned the right composition of the team proved to be very important. The team needs to contain members with deep knowledge on the data source, members that have a good understanding of the OMOP CDM and the vocabularies, and experts to implement the ETL.

Improving the interoperability of health data requires standardizing both the structure (syntactic interoperability) and the terminologies (semantic interoperability). The surveys revealed that mapping from the source structure to the standardized clinical tables of the OMOP CDM was not seen as difficult by the teams but mapping source codes to the OHDSI Standardized Vocabularies was frequently mentioned as a challenging aspect of the ETL process. This is aligned with previous literature on ETL development.^18–32^ For example, a recent publication from Oja et al.^18^ highlighted the difficulty in selecting the correct concept when there are multiple options, and issues around different levels of granularity when mapping source codes to target codes. Vocabulary mappings require medical expertise and in-depth knowledge about both the source vocabulary and the target vocabulary. Also, knowing when to stop mapping terminology is key to not wasting time, often a select subset of terms will make up the majority of the database record thus all terms do not need to be mapped.^29^ There is clear value in additional training on the mapping strategy and compliance with the OMOP conventions.

Furthermore, the vocabulary mapping process needs to be supported by tools and quality control steps. The Usagi mapping tool developed by the OHDSI community was much appreciated by the teams but there is room for further improvement, eg, to add support for collaborative mapping in which multiple members can review and approve results. As a response to the lessons learned from this study, the EHDEN consortium and OHDSI community have recently started work to improve vocabulary management, quality control, and mapping. EHDEN, for example, has implemented collaboration functionality in Usagi and OHDSI collaborators are developing new tools to support vocabulary mapping (eg, Perseus,^33^ Susana^34^).

Due to the pandemic, the ETL was supported by the EHDEN Taskforce through many online meetings. These meetings were where most of the ETL process measures were captured and were important to provide guidance on ETL design and source vocabulary mappings. Clearly, a more effective approach is to hold a multi-day face-to-face meeting with all the team members. Based on past experience this would have allowed us to design the ETLs in a much shorter time (eg, the Integrated Primary Care Information database ETL was designed in 1 month^35^).

Implementing an ETL also requires preparation in terms of data access and personnel resources. Data access delays were very common and captured through the ETL process measures. For 1 DP, it took over 6 months to get institutional review board approval to standardize their data. No progress could be made before data access was allowed. Furthermore, some DPs required a considerable amount of time to get started with the ETL design and implementation due to challenges in forming an appropriate team. For example, one team was made up entirely of physicians with no technical background, making it extremely challenging to initiate a technical task such as developing an ETL. Once a technical expert was added to the team considerable progress was made. Availability of team members was limited especially for those institutions that were severely impacted by COVID-19. The support network of certified Small to Medium-sized Enterprise (SMEs) created by the EHDEN project could be a good alternative for organizations that do not have all competences in house.

Previous papers discussing ETL transformation^1^^,^^18–20^^,^^23^^,^^28^^,^^29^^,^^32^ have seen the need for appropriate resources and appropriate access to data. For example, Overhage et al.^32^ saw the need for having the appropriate people and access to data and stated, “Each partner utilized a number of people with a wide range of expertise and skills to complete the project, including project managers, medical informaticists, epidemiologists, database administrators, database developers, system analysts/programmers, research assistants, statisticians, and hardware technicians.” Similar recommendations were made by other studies,^1^^,^^18^^,^^20^^,^^23^^,^^28^^,^^29^ and a similar recommendation can even be found in the Book of OHDSI.^4^ Having sufficient access to data before beginning ETL construction, however, has received less attention in prior research. Only 2 recent publications, Oja et al.^18^ and Yu et al.,^19^ discuss obtaining permissions to utilize the data. As we have shown, obtaining correct approval can affect the duration of the ETL development and should be considered early on.

In this work, we successfully transformed 25 different databases into the OMOP CDM using a standard process and toolkit. Despite different geographies, data types, source vocabularies, and population sizes, as well as different team compositions with differing expertise, the consistent workflow used by EHDEN proves appropriate to support the successful standardization of observational data across Europe. Across the 25 successful transformations, we continue to solidify the notions that having the appropriate persons present for the ETL and vocabulary mapping can be a challenging aspect, and in addition, we learned that groups should proactively prepare for data governance issues. This effort should provide guidance for future DPs to standardize to the OMOP CDM and participate in distributed networks. The OHDSI community must continue to evaluate and provide guidance and support for what ultimately develops the backbone of how community members generate evidence.

## Supplementary Material

ocad214_Supplementary_Data

## Data Availability

The data described above will be shared on reasonable request to the corresponding author.
