# Supplementary material for: European Health Data & Evidence Network—learnings from building out a standardized international health data network
Source: J Am Med Inform Assoc. 2023 Nov 10;31(1):209–19. doi: 10.1093/jamia/ocad214 (PMC10746315; doi:10.1093/jamia/ocad214)
Supplement: ocad214_Supplementary_Data [file ocad214_supplementary_data.docx]

Supplement for

European Health Data & Evidence Network – Learnings from Building out a Standardized International Health Data Network

Erica A. Voss^1, 2, 3^, Clair Blacketer^1, 2, 3^, Sebastiaan van Sandijk^1,4^, Maxim Moinat^1, 2^, Michael Kallfelz^1,4^, Michel Van Speybroeck^3^, Dani Prieto-Alhambra^1, 2, 6^, Martijn J. Schuemie^1, 3, 5^, Peter R Rijnbeek^1, 2^

1. OHDSI Collaborators, Observational Health Data Sciences and Informatics (OHDSI), New York, NY, USA
2. Department of Medical Informatics, Erasmus University Medical Center, Rotterdam, NL
3. Janssen Pharmaceutical Research and Development LLC, Titusville, NJ, USA
4. Odysseus Data Services, Prague, CZ
5. Department of Biostatistics, University of California, Los Angeles, Los Angeles, CA, USA
6. Centre for Statistics in Medicine, NDORMS, University of Oxford, Oxford, UK

**Keywords:** OMOP common data model, observational data, data standardization

**Issue Section:** Research and Applications

# APPENDIX 1 - DATABASE DETAILS

All of this information is taken from the publicly available EHDEN Catalog (https://portal.ehden.eu/).

## APHM - Health Data Warehouse of Assistance Publique - Hopitaux de Marseille

<http://fr.ap-hm.fr/>

The Assistance Publique – Hôpitaux de Marseille (AP-HM) is a public university hospital system with 4 hospitals, 3,400 beds and more than 12,000 health care professionals. The AP-HM is one the largest health centre in France (after Paris and Lyon). For adults and children, the AP-HM provide hospital care services going from primary care to cutting-edge treatments of complex and rare pathologies. Approximately 300,000 hospitalizations are recorded every year at the APHM, involving approximately 210,000 patients. Our information system includes multiple data sources with electronic medical record (Axigate, Cimaise), treatment prescription and deliverance (Pharma), oncology treatment (Chimio), Biology, imaging, Research (Redcap for cohorts), PMSI (Programme de Médicalisation des Systèmes d’Information). The PMSI is the French medico-administrative database for all hospitalizations based on diagnosis related-groups (DRG) that we can group into significant diagnostic categories. All the stays are coded using the International Classification of Disease (ICD-10th version). All these data are collected and stored for more than 10 years with more than 1 billion pieces of data.

Research examples:

- Fond, G., Pauly, V., Orleans, V., Antonini, F., Fabre, C., Sanz, M., Klay, S., Jimeno, M. T., Leone, M., Lancon, C., Auquier, P., & Boyer, L. (2021). Increased in-hospital mortality from COVID-19 in patients with schizophrenia. *L'Encephale*, *47*(2), 89–95. <https://doi.org/10.1016/j.encep.2020.07.003>
- Fond, G., Pauly, V., Leone, M., Llorca, P. M., Orleans, V., Loundou, A., Lancon, C., Auquier, P., Baumstarck, K., & Boyer, L. (2021). Disparities in Intensive Care Unit Admission and Mortality Among Patients With Schizophrenia and COVID-19: A National Cohort Study. *Schizophrenia bulletin*, *47*(3), 624–634. <https://doi.org/10.1093/schbul/sbaa158>
- Jaotombo, F., Pauly, V., Auquier, P., Orleans, V., Boucekine, M., Fond, G., Ghattas, B., & Boyer, L. (2020). Machine-learning prediction of unplanned 30-day rehospitalization using the French hospital medico-administrative database. *Medicine*, *99*(49), e22361. https://doi.org/10.1097/MD.0000000000022361

## APUM - Azienda Policlinico Universitaria di Modena

<https://aou.mo.it>

Since the presentation of the index case with COVID-19 in Modena city, the Azienda Policlinico Universitaria of Modena developed a Hospital electronic data record in which all consecutive patients which tested positive for COVID-19 and either are admitted to hospital or are monitored for symptoms at home. All these data are prospectively recorded in a patient electronic Chart. This chart is integrated with data obtained from laboratory systems and collects relevant clinical, biochemical and microbiological data. All therapies are collected in electronic format. Also, this chart offers an opportunity to collect data regarding frailty, metabolic alterations (such as non-alcoholic fatty liver disease) or radiological findings on ultrasound, X-ray or computed tomography that may be relevant features involved in clinical presentation or pathogenetic mechanisms of COVID-19.

Research examples:

- Guaraldi, G., Milic, J., Cesari, M., Leibovici, L., Mandreoli, F., Missier, P., Rozzini, R., Cattelan, A. M., Motta, F., Mussini, C., & Cossarizza, A. (2022). The interplay of post-acute COVID-19 syndrome and aging: a biological, clinical and public health approach. *Ageing research reviews*, *81*, 101686. <https://doi.org/10.1016/j.arr.2022.101686>
- Mussini, C., Cozzi-Lepri, A., Meschiari, M., Franceschini, E., Burastero, G., Faltoni, M., Franceschi, G., Iadisernia, V., Volpi, S., Dessilani, A., Gozzi, L., Conti, J., Del Monte, M., Milic, J., Borghi, V., Tonelli, R., Brugioni, L., Romagnoli, E., Pietrangelo, A., Corradini, E., … Guaraldi, G. (2023). Do All Critically Ill Patients with COVID-19 Disease Benefit from Adding Tocilizumab to Glucocorticoids? A Retrospective Cohort Study. *Viruses*, *15*(2), 294. <https://doi.org/10.3390/v15020294>
- Guaraldi, G., Meschiari, M., Cozzi-Lepri, A., Milic, J., Tonelli, R., Menozzi, M., Franceschini, E., Cuomo, G., Orlando, G., Borghi, V., Santoro, A., Di Gaetano, M., Puzzolante, C., Carli, F., Bedini, A., Corradi, L., Fantini, R., Castaniere, I., Tabbì, L., Girardis, M., … Mussini, C. (2020). Tocilizumab in patients with severe COVID-19: a retrospective cohort study. *The Lancet. Rheumatology*, *2*(8), e474–e484. https://doi.org/10.1016/S2665-9913(20)30173-9

## AUMC - Pacmed Data Warehouse at Amsterdam University Medical Center

<https://www.vumc.nl/>

Database contains data about COVID-19 patients collected from 25 ICUs in the Netherlands. Data includes: Conditions, Drugs, Measurements, Observations, and some Procedures.

Research examples:

- Fleuren, L. M., de Bruin, D. P., Tonutti, M., Lalisang, R. C. A., Elbers, P. W. G., & Dutch ICU Data Sharing Collaborators (2021). Large-scale ICU data sharing for global collaboration: the first 1633 critically ill COVID-19 patients in the Dutch Data Warehouse. Intensive care medicine, 47(4), 478–481. <https://doi.org/10.1007/s00134-021-06361-x>
- Fleuren, L. M., Dam, T. A., Tonutti, M., de Bruin, D. P., Lalisang, R. C. A., Gommers, D., Cremer, O. L., Bosman, R. J., Rigter, S., Wils, E. J., Frenzel, T., Dongelmans, D. A., de Jong, R., Peters, M., Kamps, M. J. A., Ramnarain, D., Nowitzky, R., Nooteboom, F. G. C. A., de Ruijter, W., Urlings-Strop, L. C., … Elbers, P. W. G. (2021). The Dutch Data Warehouse, a multicenter and full-admission electronic health records database for critically ill COVID-19 patients. Critical care (London, England), 25(1), 304. https://doi.org/10.1186/s13054-021-03733-z

## BIOCRUCES - Biocruces Bizkaia Health Research Institute

[www.biocrucesbizkaia.org](http://www.biocrucesbizkaia.org)

Anonymized data from the Electronic Medical Records from the Integrated Service Organization (ISO) Ezkerraldea Enkarterri Cruces that comprises one tertiary hospital and all the related primary care centres. Includes hospital and outpatient data collected routinely in the clinical practice, both structured and unstructured information, extracted using a free text analysis tool(with natural language processing).

## CC_NIS - University Clinical Center Nis

<http://www.kcnis.rs/>

Data is provided from almost 3400 patients from the south and eastern region of Serbia. Approximately 45% of these patients are from Niš, the rest are from the other hospital centers in the region. The data source has over 3400 patients who required hospital treatment.

## CC_SERBIA - University Clinical Center of Serbia

<http://www.kcs.ac.rs/>

University Clinical Center of Serbia, based in Belgrade, is the largest health care institution in Serbia and in the region. Heliant Health is a Health information system (HIS), with capabilities of EHR (electronic health record) and follows many hospital business processes, from patient care, financial information, to drug and material maintenance.

The data source represents an integrated information system in which close to 8000 healthcare professionals are working every day. All types of care and all specializations are provided in this institution. It covers patients from all over Serbia and the Balkans region.

Research examples:

- Paling, F. P., Troeman, D. P. R., Wolkewitz, M., Kalyani, R., Prins, D. R., Weber, S., Lammens, C., Timbermont, L., Goossens, H., Malhotra-Kumar, S., Sifakis, F., Bonten, M. J. M., & Kluytmans, J. A. J. W. (2017). Rationale and design of ASPIRE-ICU: a prospective cohort study on the incidence and predictors of Staphylococcus aureus and Pseudomonas aeruginosa pneumonia in the ICU. BMC infectious diseases, 17(1), 643. <https://doi.org/10.1186/s12879-017-2739-4>

## CPRD_AURUM - Clinical Practice Research Datalink – AURUM + Hospital Episode Statistics Admitted Patient Care (HES APC) data

<https://www.cprd.com/>

Clinical Practice Research Datalink (CPRD) is a real-world research service supporting retrospective and prospective public health and clinical studies. CPRD research data services are delivered by the Medicines and Healthcare products Regulatory Agency with support from the National Institute for Health and Care Research (NIHR), as part of the Department of Health and Social Care.

CPRD collects anonymized patient data from a network of GP practices across the UK. Primary care data are linked to a range of other health related data to provide a longitudinal, representative UK population health dataset. The data encompass 60 million patients, including 18 million currently registered patients.

In the European Health Data & Evidence Network COVID-19 Rapid Collaboration Call, the grant awarded was to support converting the hospital episode statistics admitted patient care (HES APC) data [1]. This work followed a previously awarded grand to convert the CPRD_AURUM data.

Research examples:

- Research using CPRD data has informed drug safety guidance and clinical practice and resulted in over 3,000 peer-reviewed publications. The CPRD bibliography can be downloaded from: <https://cprd.com/bibliography>.

References:

[1] CPRD linked data: HES Admitted Patient Care data. Secondary CPRD linked data: HES Admitted Patient Care data [web page]. https://cprd.com/cprd-linked-data#HES%20Admitted%20Patient%20Care%20data.

## CSS – Center for Surgical Science

<https://centerforsurgicalscience.dk/>

The Danish Colorectal Cancer Group Database (DCCG) is a national clinical quality database that has been gathering data on patients with primary colorectal cancer. The database also includes data on operations, radiological and oncological treatment and a range of pathological variable. The aim of DCCG is to improve the prognosis for patients with colorectal cancer, also by optimizing and unifying diagnostics, surgical and oncological treatment of this group of patients. Reporting to DCCG is done using the Clinical Measurement System (KMS), supplemented with data from the National Patient Register, the Pathology Register and the CPR Register.

## DATALOCH – DataLoch from University of Edinburgh

<https://dataloch.org/>

The DataLoch EHDEN database contains consecutive person records for those tested for COVID-19 after March 2020, whether in a hospital or community test setting. This has been linked to historical coded data for hospitalizations, drug prescriptions, laboratory results and deaths. This covers a geographical area in Southeast Scotland with a population of approximately 900k individuals.

Research examples:

- Lee, K. K., Doudesis, D., Ross, D. A., Bularga, A., MacKintosh, C. L., Koch, O., Johannessen, I., Templeton, K., Jenks, S., Chapman, A. R., Shah, A. S. V., Anand, A., Perry, M. R., Mills, N. L., & DataLoch COVID-19 Collaboration (2021). Diagnostic performance of the combined nasal and throat swab in patients admitted to hospital with suspected COVID-19. BMC infectious diseases, 21(1), 318. https://doi.org/10.1186/s12879-021-05976-1

## FIIBAP - Fundación para la Investigación e Innovación Biosanitaria en Atención Primaria

<https://www.fiibap.org/>

Our data sources for the extraction of relevant information in COVID-19 are: • Healthcare systems of Madrid Primary Care. • Healthcare systems (Hospitalization and Emergency) of Hospital Universitario 12 de Octubre. The information domains covered for COVID-19 are: demographics, visits, diagnoses, comorbidity, labor absenteeism, procedures, prescriptions and non-pharmacological orders, and relevant clinical and laboratory findings (included COVID-19 tests).

## FINCB - Fondazione IRCCS Istituto Neurologico Carlo Besta

<https://www.istituto-besta.it/>

766 individuals suspected to carry SARS-COV-2 and admitted to the General Hospital of Crema, Italy, between February 21st and March 13rd 2020 and followed-up until March 19th, 2020. All individuals underwent clinical and laboratory exams, SARS-COV-2 reverse transcriptase-polymerase chain reaction on nasopharyngeal swab, and chest X-ray and/or computed tomography (CT). Data on onset, course, comorbidities, number of drugs including angiotensin converting enzyme (ACE) inhibitors and angiotensin-II-receptor antagonists (sartans), follow-up swab, pharmacological treatments, non-invasive respiratory support, ICU admission, and deaths were recorded.

Research examples:

- Gili, T., Benelli, G., Buscarini, E., Canetta, C., La Piana, G., Merli, G., Scartabellati, A., Viganò, G., Sfogliarini, R., Melilli, G., Assandri, R., Cazzato, D., Rossi, D. S., Usai, S., Caldarelli, G., Tramacere, I., Pellegata, G., & Lauria, G. (2021). SARS-COV-2 comorbidity network and outcome in hospitalized patients in Crema, Italy. PloS one, 16(3), e0248498. <https://doi.org/10.1371/journal.pone.0248498>
- Buscarini, E., Manfredi, G., Brambilla, G., Menozzi, F., Londoni, C., Alicante, S., Iiritano, E., Romeo, S., Pedaci, M., Benelli, G., Canetta, C., La Piana, G., Merli, G., Scartabellati, A., Viganò, G., Sfogliarini, R., Melilli, G., Assandri, R., Cazzato, D., Rossi, D. S., … Lauria, G. (2020). GI symptoms as early signs of COVID-19 in hospitalised Italian patients. Gut, 69(8), 1547–1548. <https://doi.org/10.1136/gutjnl-2020-321434>
- Canetta, C., Accordino, S., Buscarini, E., Benelli, G., La Piana, G., Scartabellati, A., Viganò, G., Assandri, R., Astengo, A., Benzoni, C., Gaudiano, G., Cazzato, D., Rossi, D. S., Usai, S., Tramacere, I., & Lauria, G. (2020). Syncope at SARS-CoV-2 onset. Autonomic neuroscience : basic & clinical, 229, 102734. <https://doi.org/10.1016/j.autneu.2020.102734>, G. (2021). SARS-COV-2 comorbidity network and outcome in hospitalized patients in Crema, Italy. PloS one, 16(3), e0248498. <https://doi.org/10.1371/journal.pone.0248498>

## FPIO - Fondazione Poliambulanza Istituto Ospedaliero

<http://www.poliambulanza.it>

Since the beginning of the COVID-19 outbreak in Brescia (20/02/2020), Fondazione Poliambulanza has collected data from its EMR (electronic medical record) related to all patients admitted into the hospital with symptoms attributable to COVID-19. Patients have been admitted into emergency department or directly into a medical unit or into ICU and have been included regardless of the outcome of the test for COVID-19. Depending on the acuity rate, those patients have been treated into the hospital or monitored for symptoms at home. All specific relevant data have been collected from administrative records, relevant clinical notes, vital signs, laboratory results, therapies, radiological reports (ultrasound, X-ray and computed tomography).

## HDH - Health Data Hub

<https://www.health-data-hub.fr/>

The SNDS (National Health Data System) comprehensively collects individual characteristics for almost the entire French population since 2006, outpatient healthcare prescriptions and procedures reimbursed, inpatient visit, rehabilitation data and psychiatry data. It includes information on the presence of long-term chronic diseases (LTD) eligible for 100% reimbursement of healthcare expenditures, when requested by a patient's general practitioner (GP). All of this information is linked to data concerning public and private hospital stays. Hospital stay diagnoses and LTD diagnoses are coded according to the International Classification of Diseases 10th revision (ICD 10). Causes of death (ICD 10) are collected by the Epidemiological Center for the Medical Causes of Deaths (Inserm-CépiDc) and itemized in the SNDS.

Open collaborative documentation : <https://documentation-snds.health-data-hub.fr/>

Interactive dictionary : <https://health-data-hub.shinyapps.io/dico-snds/>

Support forum : <https://entraide.health-data-hub.fr/>

Algorithms on the SNDS : <https://documentation-snds.health-data-hub.fr/formation_snds/programmes.html>

ETL documentation : <https://documentation-snds.health-data-hub.fr/omop/documentation_etl/>

ETL script : <https://gitlab.com/healthdatahub/snds_omop>

Research examples:

- Semenzato, L., Botton, J., Drouin, J., Cuenot, F., Dray-Spira, R., Weill, A., & Zureik, M. (2021). Chronic diseases, health conditions and risk of COVID-19-related hospitalization and in-hospital mortality during the first wave of the epidemic in France: a cohort study of 66 million people. The Lancet regional health. Europe, 8, 100158. <https://doi.org/10.1016/j.lanepe.2021.100158>
- Thereaux, J., Lesuffleur, T., Czernichow, S., Basdevant, A., Msika, S., Nocca, D., Millat, B., & Fagot-Campagna, A. (2019). Long-term adverse events after sleeve gastrectomy or gastric bypass: a 7-year nationwide, observational, population-based, cohort study. The lancet. Diabetes & endocrinology, 7(10), 786–795. <https://doi.org/10.1016/S2213-8587(19)30191-3>
- Scailteux, L. M., Droitcourt, C., Balusson, F., Nowak, E., Kerbrat, S., Dupuy, A., Drezen, E., Happe, A., & Oger, E. (2019). French administrative health care database (SNDS): The value of its enrichment. Therapie, 74(2), 215–223. https://doi.org/10.1016/j.therap.2018.09.072

## HIC - Health Informatics Centre

<https://www.dundee.ac.uk/hic/>

Health datasets from the Tayside and Fife regions of Scotland, provided by the Health Informatics Centre (HIC) at the University of Dundee.

Research examples:

- Sullivan, F. M., Mair, F. S., Anderson, W., Armory, P., Briggs, A., Chew, C., Dorward, A., Haughney, J., Hogarth, F., Kendrick, D., Littleford, R., McConnachie, A., McCowan, C., McMeekin, N., Patel, M., Rauchhaus, P., Ritchie, L., Robertson, C., Robertson, J., Robles-Zurita, J., … Early Diagnosis of Lung Cancer Scotland (ECLS) Team (2021). Earlier diagnosis of lung cancer in a randomised trial of an autoantibody blood test followed by imaging. The European respiratory journal, 57(1), 2000670. <https://doi.org/10.1183/13993003.00670-2020>
- Walker, H., De Souza, N., Hapca, S., Witham, M. D., & Bell, S. (2020). Effect of multiple episodes of acute kidney injury on mortality: an observational study. Clinical kidney journal, 14(2), 696–703. <https://doi.org/10.1093/ckj/sfz199>
- Baird, D., De Souza, N., Logan, R., Walker, H., Guthrie, B., & Bell, S. (2020). Impact of electronic alerts for acute kidney injury on patient outcomes: interrupted time-series analysis of population cohort data. Clinical kidney journal, 14(2), 639–646. <https://doi.org/10.1093/ckj/sfaa151>

## IDIVAL - Servicio Cántabro de Salud and IDIVAL

<https://www.idival.org>

The IDIVAL database represents citizens who receive public health assistance from the Cantabrian Health Service. The information provided by Primary Care is related to annotations, diagnoses (converted from ICPC2 to SNOMED), clinical variables and vaccines. From the hospital setting, appointments, tests (SNOMED), diagnoses (converted to SNOMED from IDC10), variables and specific information on Hospital Pharmacy have been included. In both cases, laboratory results (LOINC) and electronic prescription information are included.

## IMASIS - Parc de Salut Mar Barcelona Information System

<https://www.parcdesalutmar.cat/en/>

IMASIS information system is the Electronic Health Record (EHR) system of Parc Salut Mar Barcelona (PSMar), which is a complete healthcare services organization. Currently, this information system includes and shares the clinical information of two general hospitals, one mental health care center, one social-healthcare center including emergency room settings, which are offering specific and different services in the Barcelona city area (Spain). At present, IMASIS includes clinical information from patients who have used the services of this healthcare system since 1990 and from different settings such as admissions, outpatients, emergency room and major ambulatory surgery. The database contains hospital-based information on approximately 1.5 million patients. Around 1 million of them have at least one diagnosis coded using The International Classification of Diseases ICD-9-CM and ICD-10-CM. ICD-10-CM was introduced in the system in 2018. The database includes information about patients of all ages. The average follow-up period per patient in years is 6.37 (SD±6.82). IMASIS-2 is the anonymized relational database of IMASIS, and it was created during the European Medical Information Framework (EMIF) project being the data source used for mapping to OMOP.

Research examples:

- Vogel-González, M., Talló-Parra, M., Herrera-Fernández, V., Pérez-Vilaró, G., Chillón, M., Nogués, X., Gómez-Zorrilla, S., López-Montesinos, I., Arnau-Barrés, I., Sorli-Redó, M. L., Horcajada, J. P., García-Giralt, N., Pascual, J., Díez, J., Vicente, R., & Güerri-Fernández, R. (2021). Low Zinc Levels at Admission Associates with Poor Clinical Outcomes in SARS-CoV-2 Infection. Nutrients, 13(2), 562. <https://doi.org/10.3390/nu13020562>
- Perera, G., Rijnbeek, P. R., Alexander, M., Ansell, D., Avillach, P., Duarte-Salles, T., Gordon, M. F., Lapi, F., Mayer, M. A., Pasqua, A., Pedersen, L., van Der Lei, J., Visser, P. J., & Stewart, R. (2020). Vascular and metabolic risk factor differences prior to dementia diagnosis: a multidatabase case-control study using European electronic health records. BMJ open, 10(11), e038753. <https://doi.org/10.1136/bmjopen-2020-038753>
- Reyes, C., Pistillo, A., Fernández-Bertolín, S., Recalde, M., Roel, E., Puente, D., Sena, A. G., Blacketer, C., Lai, L., Alshammari, T. M., Ahmed, W. U., Alser, O., Alghoul, H., Areia, C., Dawoud, D., Prats-Uribe, A., Valveny, N., de Maeztu, G., Sorlí Redó, L., Martinez Roldan, J., … Duarte-Salles, T. (2021). Characteristics and outcomes of patients with COVID-19 with and without prevalent hypertension: a multinational cohort study. BMJ open, 11(12), e057632. https://doi.org/10.1136/bmjopen-2021-057632

## IRCCSE - Azienda Unità Sanitaria Locale - IRCCS in Reggio Emilia

<https://www.ausl.re.it/>

Research examples:

- Salvarani, C., Dolci, G., Massari, M., Merlo, D. F., Cavuto, S., Savoldi, L., Bruzzi, P., Boni, F., Braglia, L., Turrà, C., Ballerini, P. F., Sciascia, R., Zammarchi, L., Para, O., Scotton, P. G., Inojosa, W. O., Ravagnani, V., Salerno, N. D., Sainaghi, P. P., Brignone, A., … RCT-TCZ-COVID-19 Study Group (2021). Effect of Tocilizumab vs Standard Care on Clinical Worsening in Patients Hospitalized With COVID-19 Pneumonia: A Randomized Clinical Trial. JAMA internal medicine, 181(1), 24–31. <https://doi.org/10.1001/jamainternmed.2020.6615>
- Salvarani, C., Bajocchi, G., Mancuso, P., Galli, E., Muratore, F., Boiardi, L., Catanoso, M., Pipitone, N., Cassone, G., Girolimetto, N., Croci, S., Cimino, L., Gradellini, F., Beltrami, M., Di Lernia, V., Dolci, G., Massari, M., Marata, A. M., Costantini, M., & Giorgi Rossi, P. (2020). Susceptibility and severity of COVID-19 in patients treated with bDMARDS and tsDMARDs: a population-based study. Annals of the rheumatic diseases, 79(7), 986–988. <https://doi.org/10.1136/annrheumdis-2020-217903>
- Reggio Emilia Covid-19 Working Group (2020). Prevalence of SARS-CoV-2 (Covid-19) in Italians and in immigrants in an area of Northern Italy (Reggio Emilia). Prevalenza di infezione SARS-CoV-2 (Covid-19) negli italiani e negli immigrati nell'area di Reggio Emilia. *Epidemiologia e prevenzione*, *44*(4), 304–307. <https://doi.org/10.19191/EP20.4.P304.061>

## IU - Istanbul Faculty of Medicine, Istanbul University

<https://istanbultip.istanbul.edu.tr/>

Istanbul University (IU) Istanbul Faculty of Medicine uses a bespoke EHR system ( IU- Hospital Information Management System (HBYS)) that uses ICD-10 ( International Classification of Diseases) coding. All IU hospitals' medical records are stored in a central data repository. Same as IU, all the Hospital Information System providers adopted the Turkish Ministry of Health's terminology and a common language started to be used throughout the healthcare providers in Turkey. When a citizen visits a medical doctor, all her/his healthcare information is recorded in an Electronic Health Record (EHR). ICD-10 coding is the standard for the description of illness in these records. All doctor visits, diagnostics, treatments, prescriptions, and other relevant information are entered into the record. In regard to comprehensive electronic data sources exist, Turkey's progressive approach to healthcare data infrastructure renders it ideally placed to support trials and observational studies that rely on EHR data to capture endpoints.

## LYNXCARE - LynxCare Clinical Informatics

<https://lynx.care>

A cloud data mining and natural language processing (NLP) company representing a network of Belgian hospitals gathering data around COVID-19, the network contains 10 hospitals of which 3 hospitals the data is already processed, 7 additional hospitals are currently being set up. The network is supported by Janssen Pharmaceutica NV (J&J) Bart Vannieuwenhuysen, the aim is to extend the network outside Belgium. All available in-hospital data (ADT data, clinical notes, lab data, CT reports, ..) of COVID-19 positive patients is ingested on a daily basis into a central cloud - the central LynxCare cloud processes all data (data mining/NLP) and converts both structured and unstructured data sources into OMOP CDM with SNOMED (US version), RxNorm & LOINC terminology. When starting in a center, data is ingested retroactively for all patients admitted COVID-19 positive as from 1st March 2020, all available data of these patients (also before 1st March 2020 for e.g. medical background) is ingested, on a continuous basis we receive and process new data daily, patients are also provided questionnaires (St Georges respiratory, EQ5D, symptoms, outpatient questions) for 1 year follow up as from go-live in the centers. We, LynxCare, are commissioned by these hospitals to 1) help completing the governmental minimal data registry, 2) provide clinical dashboards on top of this data to the centers, 3) process the data according to GDPR and correctly aggregate & anonymize the data to provide it for pharmaceutical research to all pharmaceutical companies (not limited to J&J who initiated this project).

Research examples:

- Van de Meulebroucke, C., Beckers, J., & Corten, K. (2019). What Can We Expect Following Anterior Total Hip Arthroplasty on a Regular Operating Table? A Validation Study of an Artificial Intelligence Algorithm to Monitor Adverse Events in a High-Volume, Nonacademic Setting. The Journal of arthroplasty, 34(10), 2260–2266. <https://doi.org/10.1016/j.arth.2019.07.039>

## MEDAMAN - Medaman Hospital Data

<https://www.medaman.be>

The data source consists of 3 parts. The main part is the so-called Minimal Hospital Data Set: a registration of all hospital stays from the 1/1/2016 with information about the patient, the stay, the diseases (ICD-10-CM) and procedures (ICD-10-PCS). The second part is a dataset with data about the medication administered during the hospital stay. The third part are data form the clinical laboratory.

Research examples:

- Byttebier, G., Belmans, L., Alexander, M., Saxberg, B. E. H., De Spiegeleer, B., De Spiegeleer, A., Devreker, N., Van Praet, J. T., Vanhove, K., Reybrouck, R., Wynendaele, E., & Fedson, D. S. (2021). Hospital mortality in COVID-19 patients in Belgium treated with statins, ACE inhibitors and/or ARBs. Human vaccines & immunotherapeutics, 17(9), 2841–2850. https://doi.org/10.1080/21645515.2021.1920271

## RCGP - Royal College of General Practitioners Research and Surveillance Centre

<https://orchid.phc.ox.ac.uk/>

ORCHID (Oxford - Royal College of General Practitioners Clinical Informatics Digital Hub) is a Primary Care Health Sciences platform developed and based at the University of Oxford. Its secure environment is in particular designed to host RCGP RSC data from a network of registered general practices (GP). In addition to weekly surveillance of infectious diseases, the large volumes of data are consolidated, customized and extracted to serve a wider audience in research collaboration, education and for quality improvement. Further information can be found in official website https://orchid.phc.ox.ac.uk/index.php/orchid-data/. Due to data quality ORCHID has achieved the label of Trusted Research Environment (UK).

Research examples:

- de Lusignan, S., Lopez Bernal, J., Zambon, M., Akinyemi, O., Amirthalingam, G., Andrews, N., Borrow, R., Byford, R., Charlett, A., Dabrera, G., Ellis, J., Elliot, A. J., Feher, M., Ferreira, F., Krajenbrink, E., Leach, J., Linley, E., Liyanage, H., Okusi, C., Ramsay, M., … Hobbs, R. (2020). Emergence of a Novel Coronavirus (COVID-19): Protocol for Extending Surveillance Used by the Royal College of General Practitioners Research and Surveillance Centre and Public Health England. JMIR public health and surveillance, 6(2), e18606. <https://doi.org/10.2196/18606>
- Hayward, G., Butler, C. C., Yu, L. M., Saville, B. R., Berry, N., Dorward, J., Gbinigie, O., van Hecke, O., Ogburn, E., Swayze, H., Bongard, E., Allen, J., Tonner, S., Rutter, H., Tonkin-Crine, S., Borek, A., Judge, D., Grabey, J., de Lusignan, S., Thomas, N. P. B., … Hobbs, F. D. R. (2021). Platform Randomised trial of INterventions against COVID-19 In older peoPLE (PRINCIPLE): protocol for a randomised, controlled, open-label, adaptive platform, trial of community treatment of COVID-19 syndromic illness in people at higher risk. BMJ open, 11(6), e046799. <https://doi.org/10.1136/bmjopen-2020-046799>
- Joy, M., Hobbs, F. D. R., McGagh, D., Akinyemi, O., & de Lusignan, S. (2021). Excess mortality from COVID-19 in an English sentinel network population. The Lancet. Infectious diseases, 21(4), e74. <https://doi.org/10.1016/S1473-3099(20)30632-0>

## SIDIAP - The Information System for Research in Primary Care

<https://pubmed.ncbi.nlm.nih.gov/35415748/>

The Information System for Research in Primary Care (SIDIAP; www.sidiap.org) is a primary care records database that covers approximately 5.8 million people, equivalent to a 74% of the population of Catalonia, North-East Spain, who are assigned to a Catalan Health Institute primary care center. It includes data since 2006 and includes high-quality data on anthropometric measurements, disease diagnoses, prescription and dispensation of drugs, laboratory tests, demographic and lifestyle information. The SIDIAP database has been linked to COVID-19 RT-PCR test results, hospital records, and regional mortality data, and mapped to the Observational Medical Outcomes Partnership (OMOP) Common Data Model (CDM) [1] . Healthcare is universal and taxpayer funded in the region, and primary care physicians are gatekeepers for all care and responsible for repeat prescriptions.

Research examples:

- Prieto-Alhambra, D., Balló, E., Coma, E., Mora, N., Aragón, M., Prats-Uribe, A., Fina, F., Benítez, M., Guiriguet, C., Fàbregas, M., Medina-Peralta, M., & Duarte-Salles, T. (2021). Filling the gaps in the characterization of the clinical management of COVID-19: 30-day hospital admission and fatality rates in a cohort of 118 150 cases diagnosed in outpatient settings in Spain. International journal of epidemiology, 49(6), 1930–1939. <https://doi.org/10.1093/ije/dyaa190>
- Recalde, M., Davila-Batista, V., Díaz, Y., Leitzmann, M., Romieu, I., Freisling, H., & Duarte-Salles, T. (2021). Body mass index and waist circumference in relation to the risk of 26 types of cancer: a prospective cohort study of 3.5 million adults in Spain. BMC medicine, 19(1), 10. <https://doi.org/10.1186/s12916-020-01877-3>
- Burn, E., Tebé, C., Fernandez-Bertolin, S., Aragon, M., Recalde, M., Roel, E., Prats-Uribe, A., Prieto-Alhambra, D., & Duarte-Salles, T. (2021). The natural history of symptomatic COVID-19 during the first wave in Catalonia. Nature communications, 12(1), 777. <https://doi.org/10.1038/s41467-021-21100-y>

Reference:

[1] Burn E, Fernández-Bertolín S, Voss EA, et al. Establishing and characterising large COVID-19 cohorts after mapping the Information System for Research in Primary Care in Catalonia to the OMOP Common Data Model. medRxiv 2021:2021.11.23.21266734 doi: 10.1101/2021.11.23.21266734[published Online First: Epub Date]|.

## UK_BIOBANK - UK Biobank

<https://www.ukbiobank.ac.uk/>

The UK Biobank is a population-level, longitudinal research study of 500,000 participants in age range 40-69 from England, Scotland and Wales recruited between 2006 - 2010. The study contains detailed baseline phenotypic (physical and biomarker measurements, diet and alcohol, cognitive function, mental health, education and employment,..), imaging (abdomen, brain and heart MRI, DXA) and genotypic (imputed genome, whole genome, exome sequencing) information. All participants have longitudinal follow-up data through electronic health records containing events from primary (231k patients) and secondary care (395k patients) and national death and cancer registration information data. Since 2012, UK Biobank has approved over 13,000 registrations from researchers working in over 1,375 institutes in 68 countries and approved over 1,200 applications to enable these researchers to access the data. Recently, the UKB has made data from national COVID-19 testing on participants (at the moment containing test results from 1,474 participants). During the current COVID-19 pandemic, the UK Biobank is releasing data from national COVID-19 testing, primary care EHR, hospital inpatient episodes, national mortality registers and Intensive care data (for participants with confirmed COVID-19) on a monthly basis.

Research examples:

- Sudlow, C., Gallacher, J., Allen, N., Beral, V., Burton, P., Danesh, J., Downey, P., Elliott, P., Green, J., Landray, M., Liu, B., Matthews, P., Ong, G., Pell, J., Silman, A., Young, A., Sprosen, T., Peakman, T., & Collins, R. (2015). UK biobank: an open access resource for identifying the causes of a wide range of complex diseases of middle and old age. PLoS medicine, 12(3), e1001779. <https://doi.org/10.1371/journal.pmed.1001779>
- Bycroft, C., Freeman, C., Petkova, D., Band, G., Elliott, L. T., Sharp, K., Motyer, A., Vukcevic, D., Delaneau, O., O'Connell, J., Cortes, A., Welsh, S., Young, A., Effingham, M., McVean, G., Leslie, S., Allen, N., Donnelly, P., & Marchini, J. (2018). The UK Biobank resource with deep phenotyping and genomic data. Nature, 562(7726), 203–209. <https://doi.org/10.1038/s41586-018-0579-z>

## ULSM - Unidade Local de Saúde de Matosinhos

<http://www.ulsm.min-saude.pt>

The Local Health Unit of Matosinhos (ULSM) is a Corporate Public Entity, integrated in the National Health Service, which provides primary, hospital, and long-term health care. It was the first Local Health Unit to be established in Portugal in 1999, and its vision is the excellence of the integrated healthcare provided to its patients, based on principles of humanity, cooperation, and innovation. The ULSM comprises the Hospital Pedro Hispano, the grouping of Matosinhos Health Centres and the Convalescence Unit. Its direct area of influence is the municipality of Matosinhos, with a population of approximately 175,000 inhabitants, spread over about 63 km2 and it is also reference to the populations of neighbors cities of Póvoa de Varzim e Vila de Conde (around 140000inhabitants). Medical specialties: Family and General Medicine, Pathological Anatomy, Radiology, Clinical Pathology, Neuroradiology, Internal Medicine, Neurology, Cardiology, Endocrinology, Dermatology, Clinical Hematology, Immunoallergology, Immunochemotherapy, Oncology, Infectious Diseases, Pneumology , Gastroenterology, Nephrology, Physical Medicine and Rehabilitation, Psychiatry, Anesthesia, Surgery, Dentistry, Ophthalmology, Orthopedics, Otolaryngology, Plastic and Reconstructive Surgery, Urology, Gynecology, Obstetrics, Pediatrics, Neonatology, Public Health. ULSM has a great experience data collection namely for clinical trials and observational studies.

Research examples:

- Sundström J, Bodegard J, Bollmann A, Vervloet MG, Mark PB, Karasik A, Taveira-Gomes T, Botana M, Birkeland KI, Thuresson M, Jäger L, Sood MM, VanPottelbergh G, Tangri N; CaReMe CKD Investigators. Prevalence, outcomes, and cost of chronic kidney disease in a contemporary population of 2·4 million patients from 11 countries: The CaReMe CKD study. Lancet Reg Health Eur. 2022 Jun 30;20:100438. doi: 10.1016/j.lanepe.2022.100438. PMID: 36090671; PMCID: PMC9459126.
- Norhammar A, Bodegard J, Vanderheyden M, et alPrevalence, outcomes and costs of a contemporary, multinational population with heart failure. Heart 2023;109:548-556.

## U_OF_TARTU - University of Tartu

<https://genomics.ut.ee/en/content/estonian-biobank>

The Estonian Biobank has established a population-based biobank of Estonia with a current cohort size of more than 200,000 individuals (genotyped with genome-wide arrays), reflecting the age, sex and geographical distribution of the adult Estonian population. Considering the fact that about 20% of Estonia's adult population has joined the program, it is indeed a database that is very important for the development of medical science both domestically and internationally.

Research examples:

- Leitsalu, L., Haller, T., Esko, T., Tammesoo, M. L., Alavere, H., Snieder, H., Perola, M., Ng, P. C., Mägi, R., Milani, L., Fischer, K., & Metspalu, A. (2015). Cohort Profile: Estonian Biobank of the Estonian Genome Center, University of Tartu. International journal of epidemiology, 44(4), 1137–1147. <https://doi.org/10.1093/ije/dyt268>

# APPENDIX 2 – SURVEY 1, 2, & 3

## Survey #1

**Research Question & Goal**

What is the impact of the composition of the team on the duration of the ETL as well as on its quality?

**Survey**

Please complete this survey on a computer with a standard desktop or laptop screen. It will be difficult to view on devices with smaller screens, such as a smart phone or tablet. The survey has been designed to operate best in Google Chrome but should also work in Firefox, Internet Explorer 11, Microsoft Edge, and Safari.

**Purpose**

Standardization to the OMOP Common Data Model (CDM) allows EHDEN to develop a federated network that can obtain real-world evidence faster and at a higher quality. You are being asked to take a survey since you are involved in converting a data source to the CDM as part of the EHDEN COVID-19 Rapid Collaboration Call. We like to have a better understanding of the expertise the team members are bringing to this data source’s conversion. Your response is very important because it will play a role in understanding what factors are critical for a successful conversion to the OMOP Common Data Model (CDM). Survey Duration

﷟This survey should take less than 10 minutes to complete.

**Confidentiality**

EHDEN will protect the privacy of your response (http://www.ehden.eu/policy-privacy/). We plan to summarize the results of this survey for your data source and all the other data sources to communicate what we learned about the rapid data conversion process. No information will be included that could identify you or your responses personally.

* Required

**About You**

1. Are you part of the local team or part of the EHDEN Taskforce? *

Mark only one oval.

- Local Team Member
- EHDEN Task Force

2. Please select which data partner you are representing? *

_________________________________________________________

3. What roles do you consider yourself experienced in? (choose 1 to many) *

*Check all that apply.*

- clinical scientist
- data manager
- informatician
- statistician
- epidemiologist
- computer scientist
- health policy
- medicine
- project manager
- other
- prefer not to say

4. What will be your primary role in this project? *

*Mark only one oval.*

- clinical scientist
- data manager
- informatician statistician
- epidemiologist
- computer scientist
- health policy
- medicine
- project manager
- other
- prefer not to say

5. How many years of experience do you have in this primary role? *

*Mark only one oval.*

- Less than 1 year
- 1 year and up to 2 years
- 2 years and up to 4 years
- 4 years and up to 5 years
- 5 or more years
- Prefer not to say

6. Realistically, how many hours a week can you dedicate to this project? *

*Mark only one oval.*

- 0-4 hours / week
- 5-8 hours / week
- 9-16 hours / week
- 17-24 hours / week
- 25-32 hours / week
- 33-40 hours / week

**Your experience with your data source**

7. How would you classify your expertise with the data source: *

*Mark only one oval.*

- Novice (minimal knowledge of the data source)
- Beginner (working knowledge of the data source)
- Competent (good working knowledge of the data source)
- Proficient (in depth understanding of the data source)
- Expert (authoritative knowledge of data source)

8. Have you personally been involved in a study leveraging this data source? *

*Mark only one oval.*

- Yes
- No

1. In a normal work setting how many hours a week do you work with this data source?

*Mark only one oval.*

- 0-9 hours/week
- 10-19 hours/week
- 20-39 hours/week
- 40 or more hours/week

**Observational research experience**

1. What is your experience with performing observational studies?

*Mark only one oval.*

- I have not participated in an observational study before.
- I have participated in an observational study.
- I have co-authored an observational study.
- I have led the design of an observational study.

**Extract, transform, and load (ETL) experience**

1. Have you converted or assisted converting a data source into a common data model or some other research model?

- No prior experience
- Some experience
- Experienced
- Very experienced
- Expert experienced

**OMOP & OHDSI experience**

12. What is your relationship with the OHDSI Community? (choose 1 to many) *

- I am new to the OHDSI Community
- I actively participate in the OHDSI meetings and work groups
- I use OHDSI tools and methods to support my research
- I have used an OMOP CDM before
- I am in the process of converting my data into the OMOP CDM
- I actively participate in discussions on the OHDSI community
- I am or have participated in an OHDSI network research study
- I have contributed code to the OHDSI GitHub

13. How long do you think it will take to complete a conversion of your data source to the OMOP Common Data Model (completion means ETL design documented, implemented, and with a built OMOP CDM)? *

*Mark only one oval.*

- 0-2 weeks
- 2-4 weeks
- 1-2 months
- 2-6 months
- >6 months

14. Please explain your thoughts around your choice on how long you think it will take to complete a conversion of your data source to the OMOP Common Data Model.

_________________________________________________________

1. How complex do you think the ETL process will be for your data source to be converted to the OMOP Common Data Model?

- Very easy
- Easy
- Neutral, neither easy nor difficult
- Difficult
- Very difficult

1. Please explain your thoughts around your choice on how complex you think it will be to complete a conversation of your data source to the OMOP Common Data Model.

_________________________________________________________

**Other**

17. The goal of this survey is to understand more about the participants converting this data source to the OMOP Common Data Model to see if composition of the team impacts the duration and quality of the CDM. Is there anything this survey did not address that you would like to share that would aid us in this understanding.

_________________________________________________________

## Survey #2

**Research Question & Goal**

What can we learn from the challenges faced during the ETL process?

**Survey**

Please complete this survey on a computer with a standard desktop or laptop screen. It will be difficult to view on devices with smaller screens, such as a smart phone or tablet. The survey has been designed to operate on the following best in Google Chrome but should also work in Firefox, Internet Explorer 11, Microsoft Edge, and Safari.

**Purpose**

You are being asked to take a survey as a team member converting a data source to the OMOP Common Data Model (CDM) as part of the COVID-19 Rapid Collaboration Call. We will be asking you and all team members involved questions to have a better understanding of the experiences they are bringing to this data source’s conversion. Your response is very important because it will play a role in understanding what factors are critical for a successful conversion to the OMOP Common Data Model (CDM). Standardization to the OMOP CDM allows EHDEN to develop a federated network that should reduce the time to provide answers in real world, health research – and no time is more fitting than now to standardize our health data so that we can facilitate open science collaboration of COVID-19 moving forward.

**Survey Duration**

This survey should take less than 25 minutes to complete.

**Confidentiality**

EHDEN will protect the privacy of your response (http://www.ehden.eu/policy-privacy/). We plan to summarize the results of this survey for your data source and all the other data sources to communicate what we learned about the rapid data conversion process. No information will be included that could identify you or your responses personally.

* Required

**General Information**

1. Please state which data partner you are representing: *

_________________________________________________________

2. What is your primary role in this process? *

- Mark only one oval.
- Technical (computer scientist, data manager, informatician, or statistician).
- Clinical (clinical scientist, epidemiologist, or medicine).
- Other.
- Prefer not to say.

3. Given where you are in the process now, how long do you think it will take to complete a conversion of your data source to the OMOP Common Data Model (completion means ETL design documented, implemented, and with a built OMOP CDM)? Measure time as total time from start to finish. *

Mark only one oval.

- 0-2 Weeks.
- 2-4 Months.
- 1-2 Months.
- 2-6 Months.
- >6 Months.

4. Please explain your thoughts around your choice on how long you think it will take to complete a conversion of your data source to the OMOP Common Data Model.

_________________________________________________________

5. Given where you are in the process now, how complex do you find or think the ETL process will be for your data source to be converted to the OMOP Common Data Model? *

Mark only one oval.

- Very easy
- Easy
- Neutral, neither easy nor difficult
- Difficult
- Very difficult

**Source Data**

6. Thinking of your source data’s tables being converted to the OMOP CDM, prior to starting this Rapid Collaboration Call how much experience did you or your team have with these tables needed in the ETL? *

Mark only one oval.

- Familiar with all the tables.
- Familiar with most of the tables.
- Familiar with some of the tables.
- Familiar with few of the tables.
- Familiar with none of the tables.
- Based on my involvement I cannot comment.

7. How many tables from your source data are in your ETL? Leave blank if you are unsure.

_________________________________________________________

8. Have you learned anything about your source data by going through this process? *

Mark only one oval.

- Yes.
- No.
- Based on my involvement I cannot comment.

9. If answered “yes” to the question above, would you care to briefly elaborate what you learned about your source data?

_________________________________________________________

10. If you answered “yes” to the question above (re: have you learned anything about your source data), do you think this insight will have impact on the process and quality of your source data in the future? _________________________________________________________

11. Do you or your team have direct access to your source data? For example, direct access could mean you have access to query your database or review individual rows of data in a file. *

Mark only one oval.

- Yes.
- No.
- Based on my involvement, I am not sure.

12. If you or your team does not have direct access to your source data could you describe what limits your direct access?

_________________________________________________________

13. To use your source data in this Rapid Collaboration Call was there any effort necessary to prepare the data? For example, did a data extract from source systems need to be put in place in order for data to be available for the ETL process? *

Mark only one oval.

- Yes, much effort was needed to prepare the raw data for ETL.
- Yes, some effort was needed to prepare the raw data for ETL.
- No, the data was in a format prior to applying to the Rapid Collaboration Call that was suitable for ETL.

**Technical Difficulties**

14. Does performing an ETL to the OMOP CDM interfere with your source data system operations? *

Mark only one oval.

- Yes, there is a large impact to our source data system when extracting data for conversion to the CDM. For example, we need to halt the operation of our source systems while the data extract occurs.
- Yes, there is a small impact to our source data system when extracting data for conversion to the CDM. For example, a small down time is needed to extract data from your source system.
- No, there is no impact to our source data system when extracting data for conversion to the CDM.
- Based on my involvement, I am not sure.

15. Are there any back-end incompatibility technology issues between the source data model and OMOP CDM that needed be considered in this ETL (e.g. differing database management systems, operating systems, or programming languages, etc.)? If there were none needed, please state “None”. If you are unsure, please leave blank.

_________________________________________________________

16. Were there any ETL operations required during this ETL (i.e. complex data extractions or natural language processing)? If so, briefly discuss below. If there were none needed, please state “None”. If you are unsure, please leave blank.

_________________________________________________________

**Knowledge Management**

17. How would you classify the documentation for your source data? What best classifies your situation? *

Mark only one oval.

- Well document, data, tables, columns, and relationships are well described.
- Documented, however some information about either data, tables, columns, and/or relationships are not well described.
- Some, but little documentation about the data, tables, columns, and/or relationships.
- No documentation exists for the source data.

18. Prior to the Rapid Collaboration call were you familiar with the OMOP Common Data Model? *

Mark only one oval.

- Yes.
- No.
- Based on my involvement, I am not sure.

19. Do you feel as sufficient information, tutorials, and support are provided for the OMOP Common Data Model? *

Mark only one oval.

- Yes.
- No.
- Based on my involvement, I am not sure.

20. Do you feel working with the EHDEN Taskforce Members have helped in your understanding of how to ETL your source data into the OMOP Common Data Model? *

Mark only one oval.

- Yes.
- No.
- Based on my involvement, I am not sure.

21. Have you found the Workshops with the EHDEN Taskforce useful? *

Mark only one oval.

- Extremely useful.
- Very useful.
- Somewhat useful.
- Slightly useful.
- Not at all useful.
- Based on my involvement, I am not sure.

22. To help us improve in the future please explain what you found useful and/or not useful.

_________________________________________________________

23. Please share any thoughts you have around how well you feel the OMOP Common Data Model is specified and how easy or hard it is to use. Additionally, if the OHDSI community were to invest in a tool or educational material to improve ease of use of the OMOP Common Data Model would there be anything you would recommend?

_________________________________________________________

24. What best describe the current state of your ETL documentation? *

Mark only one oval.

- The ETL Documentation clearly describes our ETL process.
- The ETL Documentation describes our ETL process but could be improved upon.
- The ETL documentation exists but lacks detail. T
- he ETL Documentation does not exist.

25. In the Rapid Collaboration Call, the EHDEN Taskforce had asked teams to scan their data with White Rabbit and build ETL Documentation with Rabbit in a Hat. Please share your thoughts on this process. What did you like about it? What could be improved upon?

_________________________________________________________

26. Check off if there are portions of your ETL processing that you cannot or are unwilling to share:

Check all that apply.

- ETL Code.
- Source code mappings.
- Source Data documentation.
- ETL Documentation.

27. How many source vocabularies within your source data needed to be mapped to the OMOP Vocabulary? Our source data used terms that did not already exist in the OMOP Vocabulary so a mapping exercise was performed. Leave blank if you are unsure.

_________________________________________________________

28. How confident are you in your ability to have accurate mappings between the source terminologies and the OMOP Vocabulary: *

- Mark only one oval.
- Extremely confident
- Very confident
- Somewhat confident
- Slightly confident
- Not at all confident

29. Rate the difficulty of the source vocabularies within your source data: *

Mark only one oval.

- Very Complex, there were more than 3 vocabularies that required mapping, or 1 or more vocabularies required complex mappings processes (i.e. tools outside USAGI).
- Less Complex, 3 or less vocabularies needed to be mapped and only required hand mapping or USAGI.
- There were no vocabularies that required mapping.
- I’m not aware of the vocabularies mapping requirements.

**General Feedback**

30. Our goal with this survey was to learn about technical challenges you may have faced surrounding your source data, technical difficulties, or issues with documentation and knowledge management. If you have other comments about these topics or just comments in general please feel free to share.

_________________________________________________________

## Survey #3

**Research Question & Goal**

What can we learn from the challenges faced during the ETL process?

**Survey**

Please complete this survey on a computer with a standard desktop or laptop screen. It will be difficult to view on devices with smaller screens, such as a smart phone or tablet. The survey has been designed to operate on the best in Google Chrome but should also work in Firefox, Internet Explorer 11, Microsoft Edge, and Safari.

**Purpose**

You are being asked to take a survey as a team member converting a data source to the OMOP Common Data Model (CDM) as part of the COVID-19 Rapid Collaboration Call. We will be asking you and all team members involved questions to have a better understanding of the experiences they are bringing to this data source’s conversion. Your response is especially important because it will play a role in understanding what factors are critical for a successful conversion to the OMOP CDM. Standardization to the OMOP CDM allows EHDEN to develop a federated network that should reduce the time to provide answers in real world, health research – and no time is more fitting than now to standardize our health data so that we can facilitate open science collaboration of COVID-19 moving forward.

**Survey Duration**

This survey should take less than 30 minutes to complete. Some of the survey questions will be like the previous survey, this is intentional.

**Confidentiality**

EHDEN will protect the privacy of your response (http://www.ehden.eu/policy-privacy/). We plan to summarize the results of this survey for your data source and all the other data sources to communicate what we learned about the rapid data conversion process. No information will be included that could identify you or your responses personally.

* Required

1. Please state which data partner you are representing: *

_________________________________________________________

2. What is your primary role in this process? *

Mark only one oval.

- Technical (computer scientist, data manager, informatician, or statistician)
- Clinical (clinical scientist, epidemiologist, or medicine)
- Other (not either in the technical or clinical category)
- Prefer not to say

3. Now that you have reached the end of this process, what are your feelings about the duration of time it took to complete? *

Mark only one oval.

- Much quicker than anticipated; we spent less time converting our data than I originally thought it would take.
- Slightly quicker than anticipated; we spent slightly less time converting our data than I originally thought it would take.
- About what I anticipated the process would take; the time spent was about what I thought it would take.
- Took slightly longer than anticipated; we spent slightly more time converting our data than I originally thought it would take.
- Took much longer than anticipated; we spent much more time converting our data than I originally thought
- I did not or do not have a sense if this took quicker or longer than it should have.

4. Please explain your thoughts around your choice on the previous question on project duration. *

_________________________________________________________

5. Given now that you are at the end or near the end of this process, how complex did you find the ETL process was for your data source to be converted to the OMOP Common Data Model?

Mark only one oval.

- Extremely easy
- Easy
- Neutral, neither easy nor difficult
- Difficult
- Exceedingly difficult

6. Please elaborate on your response on how complex you found the ETL process.

_________________________________________________________

**Follow-Up Questions from Survey 2**

7. Have you learned anything about your source data by going through this process? *

Mark only one oval.

- Yes.
- No.
- Based on my involvement I cannot comment.

8. If answered “yes” to the question above, would you care to briefly elaborate what

you learned about your source data?

_________________________________________________________

9. Survey #2 indicated that many data partners needed significant effort to prepare their source data to be converted to the OMOP CDM (e.g., a copy of the source data needed to be prepared so that the ETL process would not run on the source data). Which statement do you most agree with? *

Mark only one oval.

- It was not clear at the beginning that data preparation would be needed, discussion about this with the EHDEN Task Force would have been helpful upfront.
- We figured out data preparation was needed during the COVID-19 conversion process; however, it was understood and do not think it requires additional insight provided from the EHDEN Task Force.
- Data preparation is a necessary step for any project requiring data, this effort was assumed prior to starting the project.
- Not applicable, our source data did not require much preparation to start with the ETL Process.

10. Could you elaborate on what data preparation was necessary, if any?

_________________________________________________________

**Code Management & Versioning**

11. Is the code developed to generate your ETL stored in some type of shared repository? In other words, do all developers (i.e. everyone involved with creating the mapping) have access to the ETL developed for this COVID-19 Rapid Collaboration Call? *

Mark only one oval.

- Yes.
- No.
- Unsure.

12. If you answered “no” to the above question, please explain why.

_________________________________________________________

13. If you answered “yes” to the above question, what best describes the way the code is shared: *

Mark only one oval.

- Publicly available Git repository
- Private Git repository (either the one provided by EHDEN or another)
- Shared file folder with code
- Other method
- Unsure

14. Does the organization who won the COVID-19 Rapid Collaboration Call grant have access to the code? Choose the answer that best represents your situation: *

Mark only one oval.

- Yes, the code is available within the organization.
- No, the code is not available within our organization, but our SME or developer has access.
- No, the code is not available to our organization for various reasons.
- Unsure.

15. Imagine that your source data recently changed significantly. It changed enough that the ETL needs to change to be able to build a new CDM. Please rate the ability for your team to make those updates: *

Mark only one oval.

- Extremely easy to update our ETL, the developers who are familiar with the code are ready to and are already tasked with maintaining this ETL.
- Easy to update our ETL, the developers who are familiar with the code are available, but we will have to wait for availability as they are not tasked with the ETL maintenance.
- Neutral, neither easy nor difficult to update our ETL, resources who can make these changes should become available eventually.
- Difficult to update our ETL, we no longer have access to the developers who worked on this; however, we can work to make resources available.
- Exceedingly difficult to update our ETL, we no longer have access to any developers who can help make this change.

16. Please explain your thoughts around the choice on your ability for your team to update the ETL considering this hypothetical change.

_________________________________________________________

17. ETL always change over time. This happens as we learn more about our source data or the source data itself changes. It also even happens as the OMOP CDM and OMOP Vocabularies change. Please choose what would best describe your current version control of your ETL code: *

Mark only one oval.

- We do not have an effective way to manage our code version. It would be hard to describe the code changes between code from today versus code found next year at this time.
- We have not implemented a way to manage versions, however while it is possible for us to do so there are no plans to implement that at this time.
- We have not implemented a way to manage versions, but plan to do that in the future.
- We have already implemented version control of our ETL and would be able to know the differences between the code from today versus code in one year.
- Unsure what our ETL code version control is.

**Data Quality**

18. There are several tools we used to assess data quality (i.e. ACHILLES, Data Quality Dashboard, CDM Inspection Report, and Catalog Export). Please select the answer that best fits your experience: *

Mark only one oval.

- Even with the tools, I did not feel like there were detailed specifications for what to assess. Even with the help from the EHDEN Task Force I did not find the tools helpful or informative.
- The tools were supportive, however without the EHDEN Task Force I am not sure if I would have been able to make sense of what to do with the information.
- The tools provided insight, and I would have been able to make improvements on my own (without the EHDEN Task Force) but it would have been difficult or slow.
- The tools were useful, with them alone I could have made most of the necessary improvements needed for my CDM.
- The tools provided an obvious way to assess data quality and I was clear how to interpret the results.
- Based on my involvement I cannot comment.

19. Please elaborate on your experience with ACHILLES, Data Quality Dashboard, CDM Inspection Report, and Catalog Export. What were some of your challenges or what did you find helpful? Your insights will help us improve these tools for future teams converting their data.

_________________________________________________________

20. Sometimes data quality problems are issues with the source system that cannot be altered or corrected by the ETL process. An example would be the source system lacks birth year information for a small subset of patients and there is no good way to derive that information from the source data. Were any source data quality problems identified during your ETL development process? *

Mark only one oval.

- Yes.
- No.
- Unsure

21. If you answered “yes” to if data quality problems with the source system were identified during the ETL process, could you briefly describe that here?

_________________________________________________________

22. Do some of these data quality issues with the source system trigger data quality alerts from the EHDEN provided data quality tools (e.g., Data Quality Dashboard, ACHILLES, CDM Inspection Report, Catalog Export Report). *

Mark only one oval.

- Yes
- No.
- Unsure.

23. If you answered “yes” to if data quality problems with the source data that continue to trigger data quality alerts from the EHDEN provided data quality tools, please briefly describe that.

_________________________________________________________

24. Would sharing the output from the Data Quality Dashboard with others be possible? *

Mark only one oval.

- Yes – it could be publicly shared.
- Yes – it could be shared in certain situations.
- No – the information has privacy sensitivity.
- No – we are not allowed to share any analysis on our data.
- No – we would rather not reveal too much information about our source system.
- No – other reason.
- Unsure.

25. Standardizing terminologies is part of converting to the OMOP Common Data Model. What best describes your situation? *

Mark only one oval.

- None of our source vocabularies were found in the OMOP Vocabularies. We needed to use mappings added to the SOURCE_TO_CONCEPT_MAP for everything.
- Most of our source vocabularies were not found in the OMOP Vocabularies.
- Some of our vocabularies were found in the OMOP Vocabulary and some needed to be mapped.
- Most of our source vocabularies were found in the OMOP Vocabularies and only a few needed to be mapped.
- All our source vocabularies were found in the OMOP Vocabularies, nothing required mapping.

26. If you needed to map source vocabularies to standard terminologies, we offered tools like USAGI or access to individuals on the EHDEN Task Force who were part of the OMOP Vocabulary team. If you took advantage of these tools or individuals could you describe what worked well and what did not work well?

_________________________________________________________

27. If some of your vocabularies are not yet fully mapped, which source vocabularies will you continue to work?

_________________________________________________________

**ETL Operations**

28. Did you experience challenges with ability to process your data into the CDM? Select all that you faced: *

Check all that apply.

- Bandwidth or connection issues during data transfers
- Long running or inefficient queries of the source data
- Source data inaccessibility issues
- Significant CPU or memory usage
- Not enough data storage or backup compacity
- Other
- No challenges were experienced

29. Of the ETL challenges you have experienced are there any that are still of concern today? Selected all that are still issues you will face: *

Check all that apply.

- Bandwidth or connection issues during data transfers
- Long running or inefficient queries of the source data
- Source data inaccessibility issues
- Significant CPU or memory usage
- Not enough data storage or backup compacity
- Other
- No challenges were experienced

30. Please elaborate on challenges you faced or continue to face. For example, what were some of the technical impediments experienced and how did you mitigate them.

_________________________________________________________

31. Please choose the statement that most accurately describes your CDM: *

Mark only one oval.

- We have transformed our COVID-19 data and our CDM will continue to only focus on COVID-19 patients.
- We have transformed our COVID-19 patient data with plans to expand that to other patients in the future.
- We have transformed all patient data we currently have access too but have plans to expand upon that as more data becomes available.
- We have transformed all patient data and no additional ETL development is necessary at this time.
- I am not sure which of these statements accurately describes our CDM.

32. Please choose the statement that most accurately describes your organization’s view on maintaining the CDM moving forward: *

Mark only one oval.

- We have made our COVID-19 CDM and will use it for analysis purposes, however
- we do not plan much future investment into its development after this call for data and
- the COVID-19 research is over.
- We have made our CDM and plan to make light updates to it moving forward. Our
- organization still needs to see the value in this development before any further
- significant investment in its maintenance is made.
- We have made our CDM and plan to improve upon it through other EHDEN calls for
- data.
- We have made our CDM and plan to improve upon it moving forward to facilitate
- our organization’s research on our medical data.
- I am not aware of my organization's thoughts on our CDM’s use moving forward.

33. Please elaborate on your organization’s view of maintaining the CDM moving forward. What would help increase support?

_________________________________________________________

34. How often do you think you will run your ETL? *

Mark only one oval.

- It will only be run once; the data is not changing, or we cannot or will not rerun.
- We will run it when there is a need. It will be updated on an ad hoc basis.
- The intervals between rebuilds will be about a year or more.
- The intervals between rebuilds will be about every six months.
- The intervals between rebuilds will be about once a quarter.
- There will be frequent rebuilds, one or more a month.
- As the data updates, the CDM updates.
- There is no current update plan.

**General Feedback**

35. What would be your recommended skills that are necessary to develop an ETL quickly and of high quality? The roles currently recommended are individuals who have medical knowledge, data experience, technical ETL expertise, and CDM Experience.

_________________________________________________________

36. Now that you are at the end of your transformation process, if you had to do it all over again, what would you have done differently this time?

_________________________________________________________

37. Please select which step in the ETL process you found the most challenging: *

Mark only one oval.

- Summarizing the Source Data with White Rabbit
- Create ETL Design
- Mapping Source Vocabulary Codes to Standardized Concepts
- Setting up an environment for the processing of the ETL
- Implement ETL
- Perform Data Quality Assessment
- Other

38. For the previous question please explain why you found this step the most challenging. We welcome any changes you would suggest (e.g., in process, training, etc.) that could help make this step less challenging.

_________________________________________________________

39. Please rate how helpful you found the EHDEN Taskforce during this process: *

Mark only one oval.

- 1 - Not Helpful
- 2
- 3
- 4
- 5 - Very Helpful

40. Please elaborate on how helpful you found the EHDEN Taskforce during your ETL process. What worked well? What did not work well?

_________________________________________________________

41. Do you feel that the EHDEN Task Force has wrongly assessed your experience in some part of the process and because of that did not explained some parts of the process well enough?

_________________________________________________________

42. The EHDEN Academy was available to you during this process. Did you take advantage of the courses? Choose the best response: *

Mark only one oval.

- Yes – I did review one or more lessons in the EHDEN Academy
- No – I did not review any lessons in the EHDEN Academy as I was already familiar
- with the material provided
- No – I did not review any lesson in the EHDEN Academy

43. What courses would you like to see added to the EHDEN Academy? Even if you are unsure what it has to offer, list course ideas that would have helped you during this process.

_________________________________________________________

44. Our goal with this survey was to learn about ETL implementation challenges you may have faced surrounding your code management and versioning, data quality, and ETL operations. If you have other comments about these topics or just in general, please feel free to share.

_________________________________________________________
